# Analysis of *Escherichia coli* Mutants with a Linear Respiratory Chain

**DOI:** 10.1371/journal.pone.0087307

**Published:** 2014-01-27

**Authors:** Sonja Steinsiek, Stefan Stagge, Katja Bettenbrock

**Affiliations:** Experimental Systems Biology, Max-Planck-Institute for Dynamics of Complex Technical Systems, Magdeburg, Germany; National Research Council of Italy (CNR), Italy

## Abstract

The respiratory chain of *E. coli* is branched to allow the cells' flexibility to deal with changing environmental conditions. It consists of the NADH:ubiquinone oxidoreductases NADH dehydrogenase I and II, as well as of three terminal oxidases. They differ with respect to energetic efficiency (proton translocation) and their affinity to the different quinone/quinol species and oxygen. In order to analyze the advantages of the branched electron transport chain over a linear one and to assess how usage of the different terminal oxidases determines growth behavior at varying oxygen concentrations, a set of isogenic mutant strains was created, which lack NADH dehydrogenase I as well as two of the terminal oxidases, resulting in strains with a linear respiratory chain. These strains were analyzed in glucose-limited chemostat experiments with defined oxygen supply, adjusting aerobic, anaerobic and different microaerobic conditions. In contrast to the wild-type strain MG1655, the mutant strains produced acetate even under aerobic conditions. Strain TBE032, lacking NADH dehydrogenase I and expressing cytochrome *bd-II* as sole terminal oxidase, showed the highest acetate formation rate under aerobic conditions. This supports the idea that cytochrome *bd-II* terminal oxidase is not able to catalyze the efficient oxidation of the quinol pool at higher oxygen conditions, but is functioning mainly under limiting oxygen conditions. Phosphorylation of ArcA, the regulator of the two-component system ArcBA, besides Fnr the main transcription factor for the response towards different oxygen concentrations, was studied. Its phosphorylation pattern was changed in the mutant strains. Dephosphorylation and therefore inactivation of ArcA started at lower aerobiosis levels than in the wild-type strain. Notably, not only the micro- and aerobic metabolism was affected by the mutations, but also the anaerobic metabolism, where the respiratory chain should not be important.

## Introduction

The respiratory chain of *Escherichia coli* consists of alternative primary dehydrogenases, different quinone species and alternative terminal oxidases (for reviews, see [1] [2]. This versatility allows the cells flexibility to gain the maximum energy under a given growth condition, from anaerobic to microaerobic and aerobic conditions, as well as with alternative electron acceptors. To be able to define and reproduce specific microaerobic conditions, a method described by Alexeeva et al. [3] was used to quantify the oxygen supply of *E. coli* cells. In glucose-limited cultures there is an inverse linear correlation between oxygen availability and excretion of the fermentation product acetate. This allows an aerobiosis scale to be defined with 100% aerobiosis being the minimal oxygen concentration needed to suppress acetate formation and 0% aerobiosis being fully anaerobic conditions, where the specific rate of acetate formation is maximal. The wild-type MG1655 and specific metabolic mutant strains were already analyzed over the aerobiosis scale [4]; amongst others, increasing aerobiosis alters the expression of genes encoding respiratory enzymes. There are two NADH-quinone oxidoreductases in the respiratory chain of *E. coli* [5]. NADH dehydrogenase I (NDH-1, encoded in the *nuo*-operon) is a proton-pump [6], [7] while NADH dehydrogenase II (NDH-2, encoded by *ndh*) is not [5]. In glucose-limited continuous cultures expression of *nuoN* (indicative of NADH dehydrogenase I) increases gradually from 0% to 100% aerobiosis, whilst expression of *ndh* is maximal under anaerobic and low microaerobic conditions [4]. The main electron flux in aerobic, glucose-limited chemostats is mediated by NADH dehydrogenase I [6], the distribution of flux between the alternative dehydrogenases is affected by the growth rate. The existence of two NADH dehydrogenases, one pumping protons and the other not, might be advantageous in enabling flexibility in dependence of the intracellular NAD(P)H/NAD(P) ratio [8]. Under certain conditions, it might be more important to maximize the growth rate rather than the growth yield (energy generation versus efficiency) [9]. Quinones serve as link between the primary dehydrogenases and the terminal oxidases [10], [11]. Under aerobic conditions, quinol oxidases transfer the reducing equivalents from the quinols to the terminal electron acceptor oxygen. The benzoquinone ubiquinone (UQ) is the main quinone under aerobic conditions, while the naphthoquinones menaquinone (MK) and demethylmenaquinone (DMK) are more abundant under anaerobic conditions [10], [12]. Nevertheless, besides ubiquinone also naphthoquinones are able to function as electron carriers in the aerobic electron transport chain, as could be shown with mutant strains [10]. The three major terminal oxidases present in *E. coli* differ in their reaction velocity and their affinity towards oxygen. While all three contribute to generate a proton-motive force, only cytochrome *bo* (Cyt bo) functions as a true proton pump [13], [14]. It has a relatively low affinity for oxygen [15], [16] and functions therefore mainly under aerobic conditions, where its expression is maximal [15], [17] [4]. The main respiratory activity in the microaerobic range is linked to cytochrome *bd*-I (Cyt *bd-I*) [3], which has a higher affinity for oxygen [13], [15], [18], [19] and is maximally expressed in the microaerobic range [17], [20] between 50 and 100% aerobiosis [4]. *E. coli* has a third terminal oxidase (cytochrome *bd*-II; Cyt *bd-II*) that is assumed to function at very low oxygen levels [21]–[24], but takes part in the aerobic respiratory chain under glucose-limited conditions as well [22]. Accordingly, transcription of *appC* is maximal in the low microaerobic range, where the other cytochrome oxidases exhibit low expression levels [4], [21]. All three quinol oxidases are able to accept electrons not only from ubiquinol, but also from naphthoquinols [25].

The most important transcriptional regulators for the metabolic response of *E. coli* towards changes in oxygen availability are the direct oxygen sensing regulator Fnr (“fumarate and nitrate reduction”) [26]–[29] and the two component regulatory system ArcBA (“anoxic redox control protein”) [30]–[32]. Under anaerobic conditions, ArcA is activated through an intermolecular phosphorylation via ArcB [33], [34]. Fnr and ArcA coordinate gene expression under aerobic and anaerobic conditions mainly by repressing those genes required for aerobic growth (e.g. coding for enzymes of the TCA cycle or respiratory chain) and activating gene expression for the fermentative metabolism, respectively anaerobic respiration [28], [35]–[38]. While Fnr is directly inactivated by oxygen [39] and therefore is mainly active under strict anaerobic conditions, the ArcBA system is active under microaerobic conditions as well [40]–[43]. Until now the nature of the signal sensed by the sensor ArcB is not completely elucidated, although the redox state of the quinone pool is discussed. An activating function of ubiquinol, the reduced form of ubiquinone, was described [44], respectively an inhibitory effect of the oxidized form [45], [46]. In addition, an effect of some metabolites (like lactate, pyruvate and acetate) in enhancing ArcB phosphorylation has been shown [34], [47], [48]. Recently, a role of the menaquinones under conditions where their occurrence is enhanced compared to ubiquinone, was elucidated [49], [50].

The existence of two NADH dehydrogenases and three quinol oxidases with different energetic efficiencies enables the flexibility to optimize the electron flux through the electron transfer chain for various growth conditions. To clarify the need for an efficiently working respiratory chain with a high H^+^/e^−^ ratio under glucose-limited conditions at different oxygen supplies, deletion strains were analyzed. Isogenic mutant strains which lack NADH dehydrogenase I but express all terminal oxidases, respectively with only one of the terminal oxidases, were examined over the aerobiosis range to analyze the effect of the deletions, especially the effect of the resulting linear respiratory chain with a fixed ratio of H^+^/e^−^ and lowered energetic efficiency. In the wild-type strain, ratios between 2 and 8H^+^/2e^−^ are possible. While strain TBE029 (Δ*nuoB*) can still achieve H^+^/2e^−^ ratios of 2-4, strains TBE031 (Δ*nuoB*, Δ*cydB*, Δ*appB*), TBE032 (Δ*nuoB*, Δ*cydB*, Δ*cyoB*) and TBE042 (Δ*nuoB*, Δ*cyoB*, Δ*appB*) have a fixed ratio of 4 (TBE031), respectively 2H^+^/2e^−^ (TBE032, TBE042).

One focus of this study was the activity of the ArcBA regulatory system in these mutant strains. Strains with a linear respiratory chain, lacking NDH-I and two of the cytochrome oxidases have already been analyzed under aerobic conditions with respect to their energetic demand and quinone content, respectively redox state in another strain background (BW25113) [22]. Nevertheless, especially the microaerobic range, where the activity of the quinol oxidases overlap and more than one contributes to the electron flow, is an interesting field to understand the fine-tuning of the metabolism to obtain the maximal benefit for *E. coli* by weighting the advantage of flexibility against maximal yield.

## Materials and Methods

### Bacterial strains and culture conditions

All strains are derivatives of *E. coli* K12 MG1655 and are listed in Table 1. Mutant strains were kindly provided by the cooperation partners from Amsterdam University (K. J. Hellingwerf). The Δ*nuoB::kan*, Δ*cyoB::kan*, Δ*cydB*::*kan* and Δ*appB*::*kan* allels were transferred by P1 transduction using strains JW5875, JW0421, JW0723 and JW0961 from the KEIO collection [51] as donor strains. The resistance cassettes were eliminated as described by Datsenko and Wanner [52].

**Table 1:** Strains used in this work.

Strain	Genotype	Residual Cytochrome Oxidase(s)	Origin/Reference
MG1655	λ^−^F^−^ *rph* ^−^1Fnr^+^	Cyt *bo*, Cyt *bd-I*, Cyt *bd-II*	
TBE029	MG1655 Δ*nuoB*	Cyt *bo*, Cyt *bd-I*, Cyt *bd-II*	A. Ter Beek
TBE031	MG1655 Δ*nuoB*, Δ*cydB*, Δ*appB*	Cyt *bo*	A. Ter Beek
TBE032	MG1655 Δ*nuoB*, Δ*cydB*, Δ*cyoB*	Cyt *bd-II*	A. Ter Beek
TBE042	MG1655 Δ*nuoB*, Δ*cyoB*, Δ*appB*	Cyt *bd-I*	A. Ter Beek

Strains were characterized in chemostat cultures with differing oxygen supply (3–4 cultivations for the wild-type at each aerobiosis value, 2 cultivations for the mutant strains at each aerobiosis value). LB_0_ precultures were used for inoculation of minimal medium precultures. The medium used was based on Evans medium [53], for precultures and batch experiments containing: 20 mM glucose, 0.05 M Na_x_H_x_PO_4_-buffer, 0.01 M KCl, 0.00125 M MgCl_2_, 0.1 M NH_4_Cl, 0.002 M Na_2_SO_4_, 0.02 mM CaCl_2_, 0.001 mM selenite, 0.38 g/l nitrilotriacetic acid and 5 ml/l trace element solution (0.412 g/l FeCl_3_, 5.4 g/l MnCl_3_, 0.172 g/l CuCl_2_, 0.476 g/l CoCl_2_, 0.064 g/l H_3_BO_3_, 0.004 g/l Na_2_MoO_4_, 10 ml/l 37% HCl) the pH was adjusted to pH 7. Aerobic cultivations were performed at 37°C in conical flasks with baffles on a rotary shaker at 250 rpm, precultures for anaerobic cultivations were not shaken.

For continuous cultivations in a stirred-tank reactor Evans medium was used with 20 mM glucose as carbon source and the pH was maintained at 6.9. The medium for bioreactor experiments was not buffered; 0.01 M NaH_2_PO_4_ was added instead of 0.05 M Na_x_H_x_PO_4_-buffer. Chemostat experiments were carried out in Infors bioreactors (Infors GmbH, Einsbach) with 400 ml working volume at a temperature of 37°C. Agitation rate was set to 550 rpm and aeration rate adjusted to 0.5 vvm (0.2 l/min) with a mixture of air and argon (argon 4.8). The aerobiosis scales for the different strains [3] were defined from measurement of acetate formation rates in continuous cultures grown at different oxygen availabilities. Due to a linear correlation between the acetate formation rate (in steady state) and oxygen concentration in the inflow for the wild-type strain MG1655, the point of 100% aerobiosis (minimal oxygen concentration to prevent acetate excretion) can be determined. The theoretical points of 120 and 150% were calculated from the linear equation. Experiments were performed at the chosen points of 0, 20, 50, 80, 100% and 120% aerobiosis (respectively 150% aerobiosis for the mutant strains). The optical density was determined at 420 nm. Cultivations were started at an optical density of 0.2 (OD_420nm_, representing ca. 10^8^ cells/ml) in batch mode and switched to continuous fermentation at OD_420nm_ = 1–2; dilution rate was set to 0.2 h^−1^. Samples for analyzing organic acids in the culture, mRNA expression rates and ArcA phosphorylation in the cells were taken after reaching a steady state for a minimum of four residence times. The establishment of a steady state was validated by observing several parameters like optical density, redox value and carbon dioxide and oxygen concentrations in the exhaust air.

### Measurement of biomass and extracellular metabolite concentrations

Dry cell weight (DCW) was estimated after centrifugation of 10 ml culture broth (20 minutes, 5.000 rpm, 4°C), resuspending the pellet in 5 ml deionised water, centrifuging (15 minutes, 5.000 rpm, 4°C) and drying the pellet for 24 hours at 105°C. For measuring extracellular metabolite concentrations, cell culture was centrifuged for 5 minutes at 13.000 rpm (4°C) and the supernatant stored at −20°C. Enzymatic test kits from Boehringer (Mannheim) r-biopharm were used for analyzing glucose, acetate, formate, succinate, ethanol and lactate. Tests (except ethanol) were adapted for use in microtiter plates. A portion of the supernatant was filtered and analyzed in a Reversed Phase HPLC: Agilent 1100 Series with a DAD detector (Agilent Technologies), column: Inertsil ODS-3 (RP-18 100A, 250×4.6 nm, Gil Science Inc.), solvent: 0.1 M NH_4_H_2_PO_4_ pH 2.6, pump rate: 1 ml min^−1^, injection volume: 10 µl. Oxygen and carbon dioxide proportions in the exhaust gas were analyzed on-line via Blue Sens gas sensor (Herten).

### Gene expression analysis by RT-PCR

Cell culture containing ∼10^9^ cells was quenched in twice the volume of mRNA-protect solution (RNAprotect Bacteria reagent, Qiagen, Hilden), vortexed for 5 seconds and incubated at room temperature for 5 minutes. After centrifugation for 5 minutes at 13000 rpm (4°C) the pellet was stored at −80°C. For RNA isolation and purification the purification kit from Epicentre (Master Pure RNA purification Kit, Madison) was used. For determining the purity and concentration of mRNA, the optical density at 260 and 280 nm was measured using a NanoDrop spectrophotometer. Transcription of isolated mRNA to cDNA was performed with the RevertAid™ H Minus First Strand cDNA Synthesis kit (Fermentas). Two samples were taken from each experiment from a minimum of two (three to four for the wild-type strain) independent cultivations (different chemostat experiments) and purified. Six mRNA aliquots were transcribed to cDNA and pooled; from these pools three RT-PCR runs were performed. Relative quantitative RT-PCR of different cDNA samples was carried out in a Rotor-Gene 6000 from Corbett life science using MESA GREEN qPCR MasterMix Plus (Eurogentec) with SYBR® Green as detection agent. A list of the investigated genes with corresponding enzymes and relevant primer pairs are listed in Table 2.

**Table 2:** List of genes and primer pairs for real-Time RT-PCR

Gene	Enzyme	Primer
*aceA*	isocitrate lyase	5′-CCTCGGCGCACTGACTGG-3′
		3′-AGTCAGCGAGATAGGCCGTT -5′
*ackA*	acetate kinase	5′-ACACCGCGTTCCACCAGACTATGC-3′
		3′-ACATTGGGTCCTTCGCCGTTTTTA -5′
*acs*	acetyl-CoA synthetase	5′-GCACCAGGCGGAAGAGATGAAC -3′
		3′-CAATAGACCACATGCGCCGCGA -5′
*appC*	cytochrome *bd-II* terminal oxidase	5′-TGACCGGGGCCATGTTTATTAT-3′
		3′-TACTTCAGCGCGTTCATGTTGC -5′
*cydA*	cytochrome *bd-I* terminal oxidase	5′-TGCGGCCTGTATACCCTGTTCC-3′
		3′-GTGCTGATGAGTCGGCCGTGC -5′
*cyoA*	cytochrome *bo* terminal oxidase	5′-CCGCTGGCACACGACGAGA-3′
		3′-ACGATGGCACTTACTTTAGCGAA -5′
*gltA*	citrate synthase	5′-GCTGGCGGCGTTCTATCAC-3′
		3′-GGTAAACAAATGGGCGCGT-5′
*ndh*	NADH dehydrogenase II	5′-GTCGATCGTAACCACAGCCA-3′
		3′-AACTCGATAGACCGGGTACG-5′
*nuoN*	NADH dehydrogenase I	5′-TGTCGCGTTGGGTAAAAACC-3′
		3′-CGGAGCCGAAGTTTGAGAGAG-5′
*poxB*	pyruvate oxidase	5′-ATCATGCGCCACAACCAGTCGT-3′
		3′-ATAACAAGTACGGGACGCGCCA -5′
*recA*	DNA strand exchange and	5′-CGCTTGGGGCAGGTGGTCT-3′
	recombination protein	3′-TTTTGGTGCGACTGCGACGT -5′
*rpoD*	sigma 70 (sigma D) factor	5′-TCTGCGTATGCGTTTCGGTATC -3′
		3′-TTGACGTGGGCTCGGCA -5′
*sdhD*	succinate dehydrogenase	5′-GATCGGTTTCTTCGCCTCTG-3′
		3′-TACACCGTCCACAACTGGC-5′
*sucA*	2-oxoglutarate dehydrogenase	5′-TCCGACACCGCGCAAAATCTAC-3′
		5′-CATCACCGTCTCACCGCAGGCT -3′
*ybhC*	outer membrane lipoprotein	5′-GTCGCGGCGCAGTGGTGTT-3′
		3′-AAGGAGCGGCATTTGTCGGCA -5′

Amplification conditions were as follows: 95°C for 10 minutes, 40 cycles at 95°C for 15 seconds and 60°C for 1 minute. A negative control without template was conducted for each gene in each PCR run, and a control for DNA-contamination was implemented by using the purified mRNA samples as template. Relative quantification and error propagation were calculated with the software qBase^PLUS^ (Biogazelle) with efficiency correlation and normalization to stably expressed reference genes [54]. *recA, rpoD* and *ybhC* were used as references, and samples were normalized to expression of the wild-type strain under anaerobic conditions, for direct comparison of the mutant expression pattern to the parent strain. Changes in gene expression levels were considered as significant when differences were at least 2-fold.

### Determination of ArcA-Phosphorylation

ArcA phosphorylation levels were determined by phos-tag™ acrylamide gel electrophoresis and Western blotting as described by Rolfe et al. [55].

## Results

The possibility to combine various NADH dehydrogenases and terminal oxidases with different efficiencies of proton translocation allows *E. coli* flexibility to deal with diverse growth conditions [6]. To clarify the need for a tuneable respiratory chain with a variable H^+^/e^−^ ratio under glucose-limited conditions at different oxygen supplies, deletion strains were analyzed. The mutant strain TBE029 lacks NDH-I, the NADH dehydrogenase which couples the electron transfer to the generation of a proton motive force. The electron transport chain is therefore less efficient and the ATP yield per oxygen molecule lower than in the wild-type strain [6]. Emanating from this parent strain, different mutant strains were constructed, which lack additionally two of the quinol oxidases. These strains thus have a linear respiratory chain with a fixed H^+^/e^−^ ratio, which is lower than in the wild-type strain. These strains were analyzed at different oxygen availabilities under glucose limiting conditions. In these cultivations the strains are hence glucose as well as oxygen limited. In a previous study an analysis of the wild-type strain MG1655 as well as of isogenic Δ*sdhC* and Δ*frdA* mutants under anaerobic, microaerobic and aerobic conditions regarding by-product formation rates and gene expression pattern in dependence of the oxygen availability has been performed [4] in order to determine the influence of specific enzyme reactions at microaerobic conditions. To be able to assign the effects resulting from the deletion of NADH dehydrogenase I to the ones emanating from the deleted quinol oxidases, some of the data of the parent strain MG1655 are shown in brief again.

**Figure 1:**
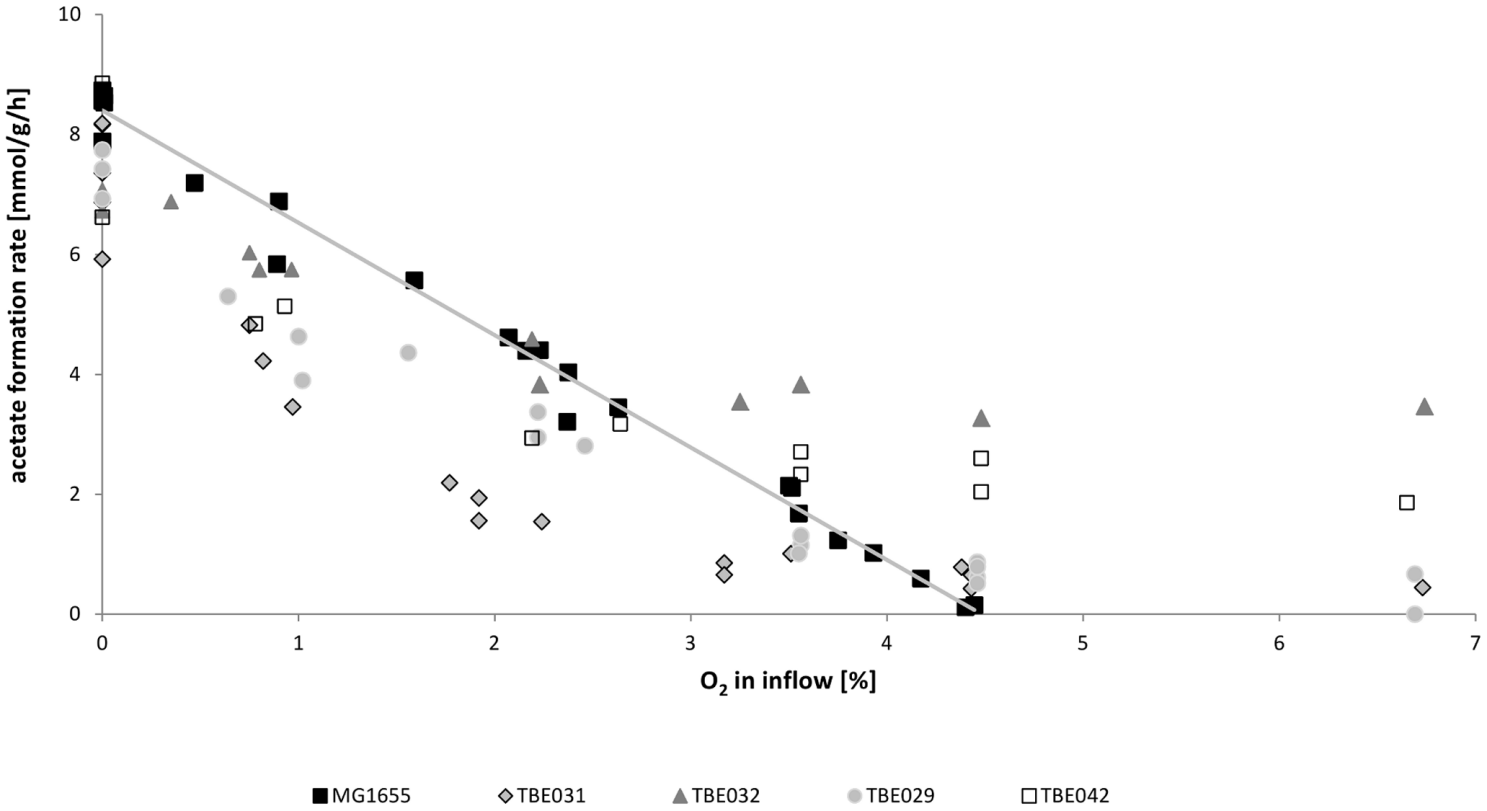
Aerobiosis scales aof MG1655 and the mutant strains. The correlation between the percentage of oxygen in the input gas and the acetate production of the different strains was analyzed in bioreactor experiments. While MG1655 displays a linear inverse correlation with no longer detectable acetate production at about 4.5% oxygen in the input gas the mutant strains showed no linear correlations and acetate production was not completely abolished even at higher O_2_ input rates.

**Figure 2:**
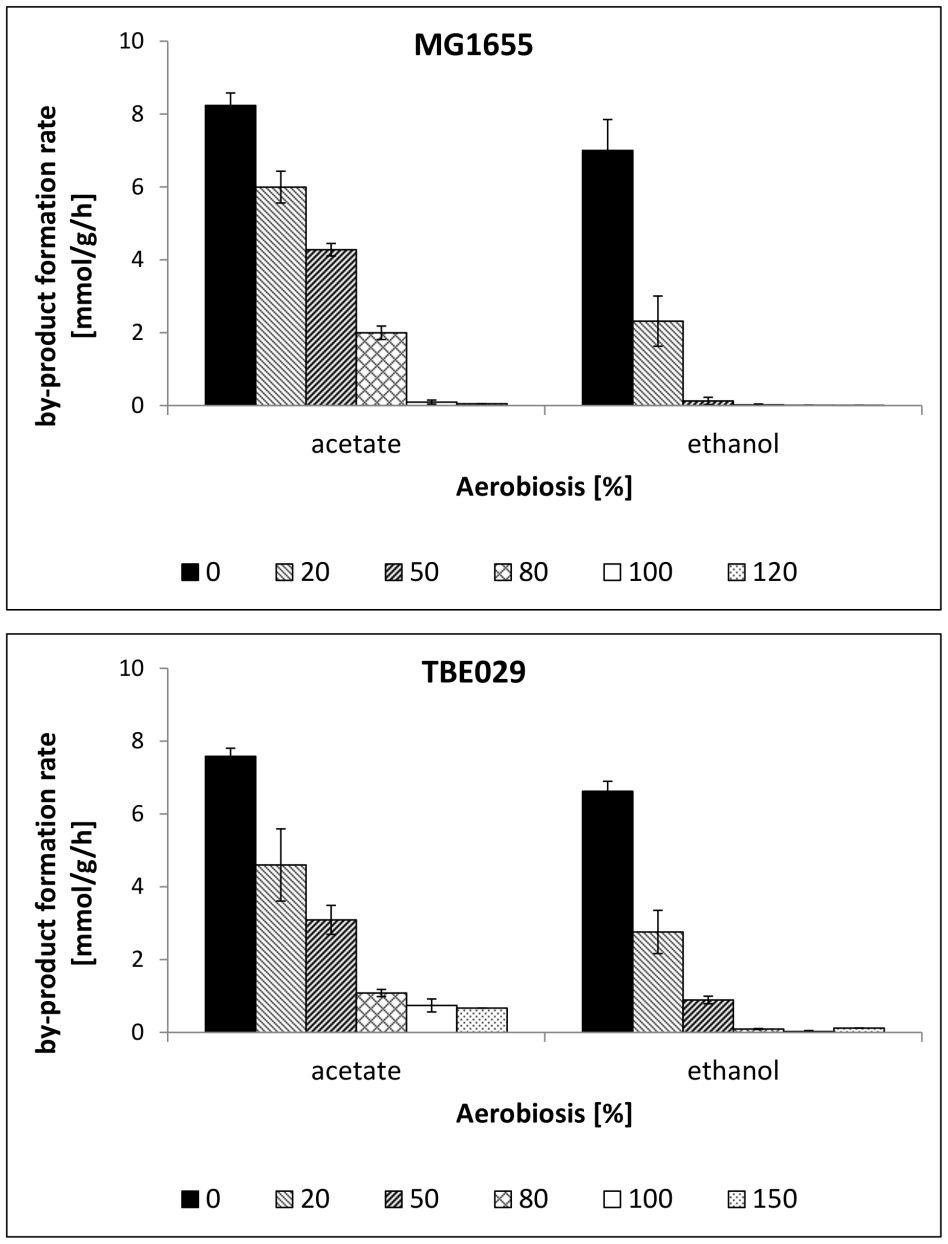
Acetate und ethanol formation rates at different oxygen concentrations in glucose-limited cultures for the wild-type strain MG1655 (1a) and strain TBE029 (1b). 100% aerobiosis corresponds to the minimal oxygen concentration in glucose-limited cultures to prevent acetate formation in MG1655. TBE029: Δ*nuoB*.

The inverse linear relationship between oxygen in the inflow and acetate formation rate in glucose-limited cultures for the wild-type strain (Fig. 1 and Fig.2a) offers the possibility of adjusting different microaerobic conditions. In this case the minimal oxygen input rate that is needed to inhibit acetate formation is set as 100% aerobiosis. Different aerobiosis levels can now be adjusted by variation of the oxygen input. This strategy allows measuring the metabolic state of cells under defined microaerobic conditions. Notably, for the wildtype the dissolved oxygen tension as can be measured by standard oxygen electrodes is zero for aerobiosis levels below 100%. The mutant strains with defects in the respiratory chain excreted the by-product acetate even under aerobic conditions (Table 3) and different to the parent strain it was not possible to further reduce acetate formation by increasing the oxygen input (Fig.1). We adopted therefore the aerobiosis scale of the wild-type strain. Accordingly, 100% aerobiosis in the mutant strains does not imply that there is no by-product formation, but corresponds to the conditions, where no by-product formation occurs at the given oxygen concentration for the wild-type, namely 4.46% oxygen in the input gas. In the following we will refer to% aerobiosis as it is more intuitively understandable than the corresponding concentration of oxygen in the input gas. For the mutants all aerobiosis points including 150% aerobiosis are theoretical points, calculated from the linear equation derived for MG1655. The mutant strains were analyzed under higher oxygen supplies to examine if acetate formation is an inherent characteristic of the mutant strains or merely terminates at higher concentrations than in the wild-type. Notably, the inverse linear correlation between oxygen input and acetate formation is not observed for the mutant strains (Fig.1) and obviously results from the interplay of the different NADH dehydrogenases and quinol oxidases. In addition, different from the wildtype strain, the mutants strains showed measurable dissolved oxygen concentrations already at aeration corresponding to 80% aerobiosis of the wildtype (Tab.S1).

**Table 3:** Acetate l formation rates from glucose-limited chemostat cultures at different aerobiosis levels.

	Acetate formation rate [mmol*g^−1^*h^−1^]			
Aerobiosis [%]	TBE029	TBE031	TBE032	TBE042
0	7,58±0,22	7,65±0,64	6,90±0,25	7,74±1,58
20	4,60±0,99	4,17±0,68	5,84±0,16	4,99±0,21
50	3,09±0,40	1,89±0,33	4,21±0,54	3,06±0,17
80	1,08±0,10	0,93±0,11	3,69±0,20	2,52±0,27
100	0,74±0,18	0,61±0,18	3,27±0,01	2,32±0,40
150	0,67±0,13	0,44±0,09	3,46±0,29	1,86±0,18

By-product formation rates are calculated per hour and gram dry cell weight [mmol*g^−1^*h^−1^]. TBE029: Δ*nuoB*; TBE031: Δ*nuoB*, Δ*cydB*, Δ*appB*; TBE032: Δ*nuoB*, Δ*cydB*, Δ*cyoB*; TBE042: Δ*nuoB*, Δ*cyoB*, Δ*appB*.

To be able to differentiate between the impact of the deletion of NADH dehydrogenase I and the changes in the metabolism based on the additional deletion of two cytochrome oxidases, the next paragraph focuses on the comparison of strain TBE029 (bearing NDH II and Cyt *bo*, Cyt *bd-I* and Cyt *bd-II*) with the wild-type strain MG1655.

### Changes based on NDH I deletion

Under anaerobic conditions (0% aerobiosis), *E. coli* MG1655 excreted besides acetate the fermentation products ethanol, formate and succinate. Lactate formation occurred only under conditions of glucose excess [4]. With increasing aerobiosis the formation rates of these by-products decreased. Deletion of NADH dehydrogenase I (strain TBE029) caused low but measurable acetate formation under conditions, where the wild-type already showed a complete aerobic metabolism without any by-product formation (100% aerobiosis) and also at higher oxygen availabilities (Fig. 2). In addition, acetate production under microaerobic conditions also seemed to be affected, as the strain excreted lower amounts of acetate between 20 and 80% aerobiosis (Fig. 1). Instead, excretion of ethanol was elevated in the mutant strain (Fig. 2). This shift in by-product synthesis might reflect a lower NADH oxidation via the respiratory chain, which is compensated by the NADH consuming ethanol production. Differences between the wild-type and TBE029 were also observable in succinate production, with TBE029 excreting higher amounts under anaerobic conditions (0% aerobiosis). None of the strains produced significant amounts of succinate at 20% (MG1655: 0.8 mmol•g^−1^•h^−1^) or higher aerobiosis.

**Figure 3:**
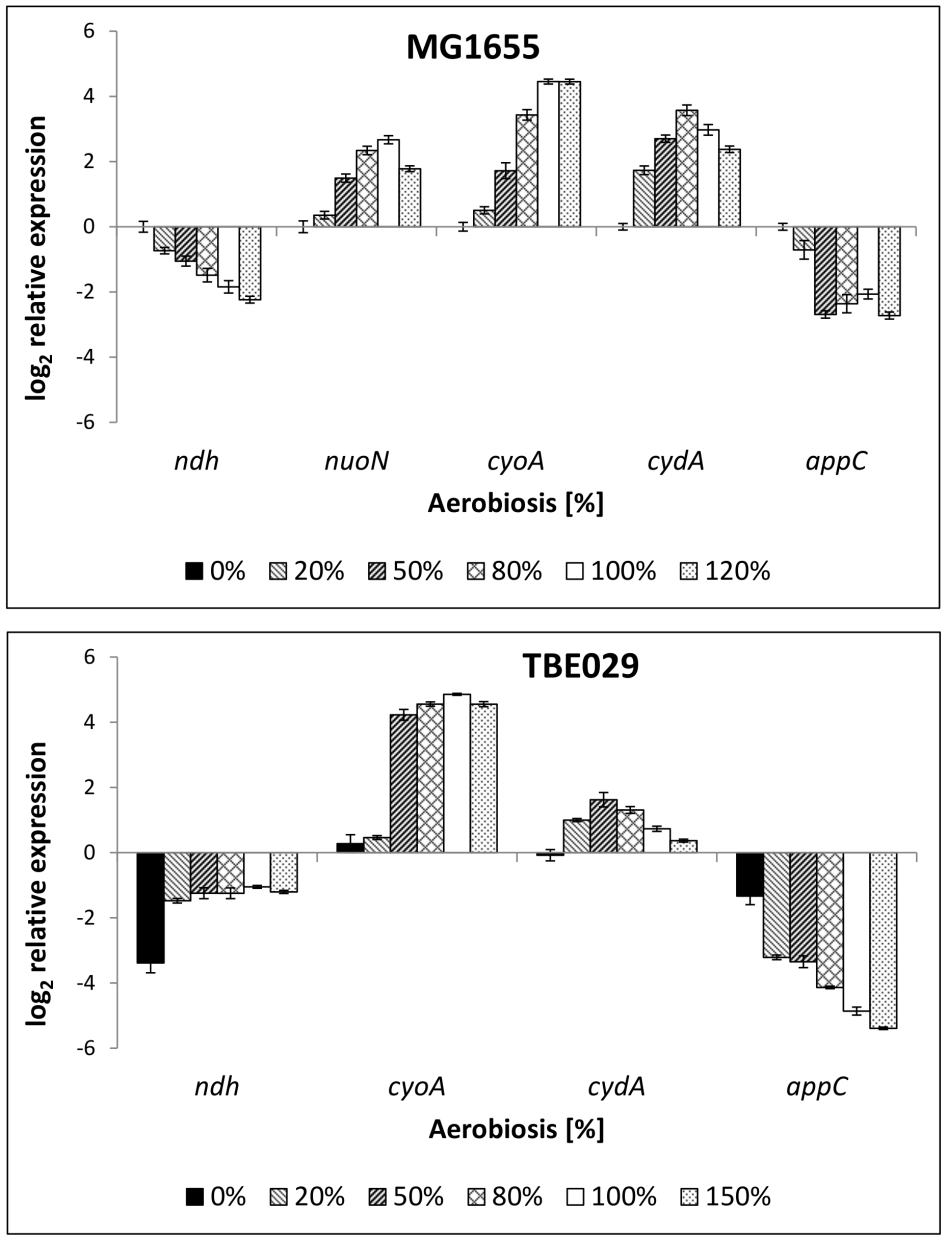
Relative expression of genes coding for respiratory enzymes in *E. coli* MG1655 (2a) and mutant strain TBE029 (2b). Transcription of NADH dehydrogenase I and II and of the cytochrome oxidases *bo*, *bd-I* and *bd-II*. The transcription pattern was analyzed in cells from glucose-limited continuous cultures and normalized to the reference genes *recA, rpoD* and *ybhC* and to the expression of the wild-type strain under anaerobic conditions. TBE029: Δ*nuoB*. *ndh*: NADH dehydrogenase II, *nuoN*: NADH dehydrogenase I, *cyoA*: subunit of cytochrome oxidase *bo*, *cydA*: subunit of cytochrome oxidase *bd-I*, *appC*: subunit of cytochrome oxidase *bd-II*.

To see if changes in the production of fermentation products are reflected in the transcription pattern of genes encoding enzymes of the aerobic and anaerobic metabolism, gene expression of selected genes (coding for enzymes of the TCA-cycle, the fermentation pathways, especially for acetate formation and also assimilation, as well as respiratory enzymes) was analyzed. Expression of *ndh*, encoding NADH dehydrogenase II, was highest at 0% aerobiosis and decreased with increasing oxygen availability in MG1655 (Fig. 3a). Its anaerobic expression was lower in TBE029, although it was the sole NADH dehydrogenase, maybe reflecting that NADH dehydrogenases are not important under fermentative conditions anyway. The transcription level between 20 and 150% aerobiosis was almost constant and did not show any dependency of oxygen availability. In the parent strain, cytochrome oxidase *bo* (*cyoA*) is maximally expressed under fully aerobic conditions, cytochrome *bd*-I oxidase (*cydA*) in the microaerobic range and cytochrome *bd*-II oxidase (*appC*) at very low oxygen concentrations [4]. The main respiratory activity below 50% aerobiosis is presumably linked to cytochrome *bd-II* oxidase with rising contribution of cytochrome *bo* oxidase with increasing oxygen availability [3]. Strain TBE029 showed an increased expression of cytochrome oxidase *bo* at 50% aerobiosis and repressed transcription of both *bd* oxidases (Fig. 3b), indicating that flux through cytochrome *bo* is enhanced, probably due to energetic demands. As the remaining NADH dehydrogenase is not contributing to the proton motive force, the use of the terminal oxidase with the highest H^+^/2e^−^ ratio might be advantageous (4 instead of 2 H^+^/2e^−^). Unlike MG1655, increase of *cyoA* expression was very steep between 20% and 50%, but rather constant between 50 and 150% aerobiosis.

**Figure 4:**
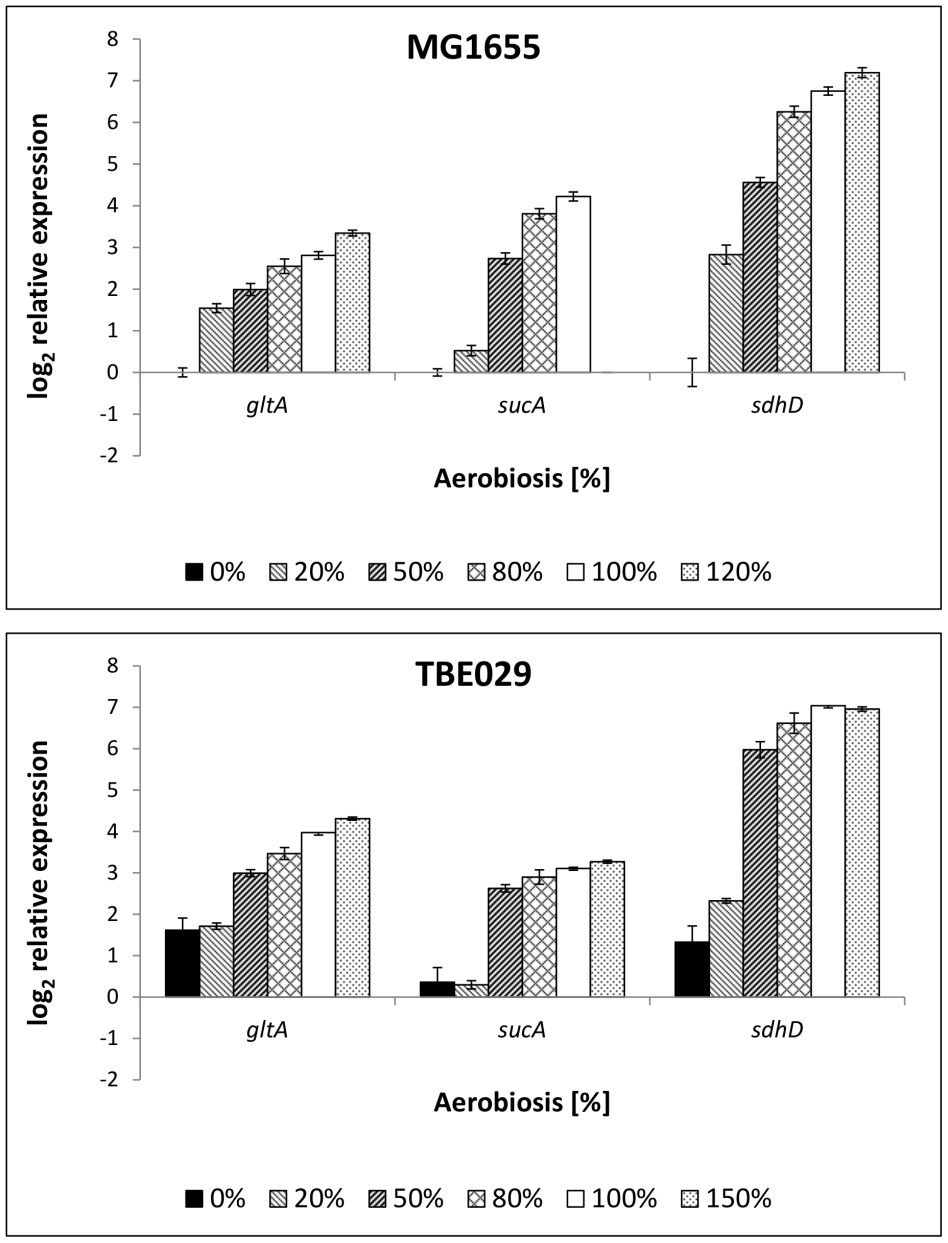
Relative expression of genes coding for enzymes of the TCA cycle in *E. coli* MG1655 (3a) and mutant strain TBE029 (3b). Transcription of citrate synthase, 2-oxoglutarate dehydrogenase and succinate dehydrogenase. The transcription pattern was analyzed in cells from glucose-limited continuous cultures and normalized to the reference genes *recA, rpoD* and *ybhC* and to the expression of the wild-type strain under anaerobic conditions. TBE029: Δ*nuoB*. *gltA*: citrate synthase, *sucA*: subunit of 2-oxoglutarate dehydrogenase, *sdhD*: subunit of succinate dehydrogenase.

The TCA cycle is repressed under anaerobic conditions and up-regulated with increasing aerobiosis in wild-type *E. coli* (Fig. 4a). The anaerobic repression of the TCA cycle was lower in TBE029 than in the parent strain, as indicated by a higher anaerobic expression of *gltA, sucA* and *sdhC* (Fig. 4b). Although no drastic changes in expression of *gltA*, *sucA* and *sdhD* became visible in the mutant, the aerobiosis profiles of these genes differed in comparison to MG1655. While in MG1655 gene expression increased gradually with increasing aerobiosis, in TB029 a steplike increase of gene expression between 20% and 50% was observed, similar to the expression of *cyoA* (Fig. 3).

**Figure 5:**
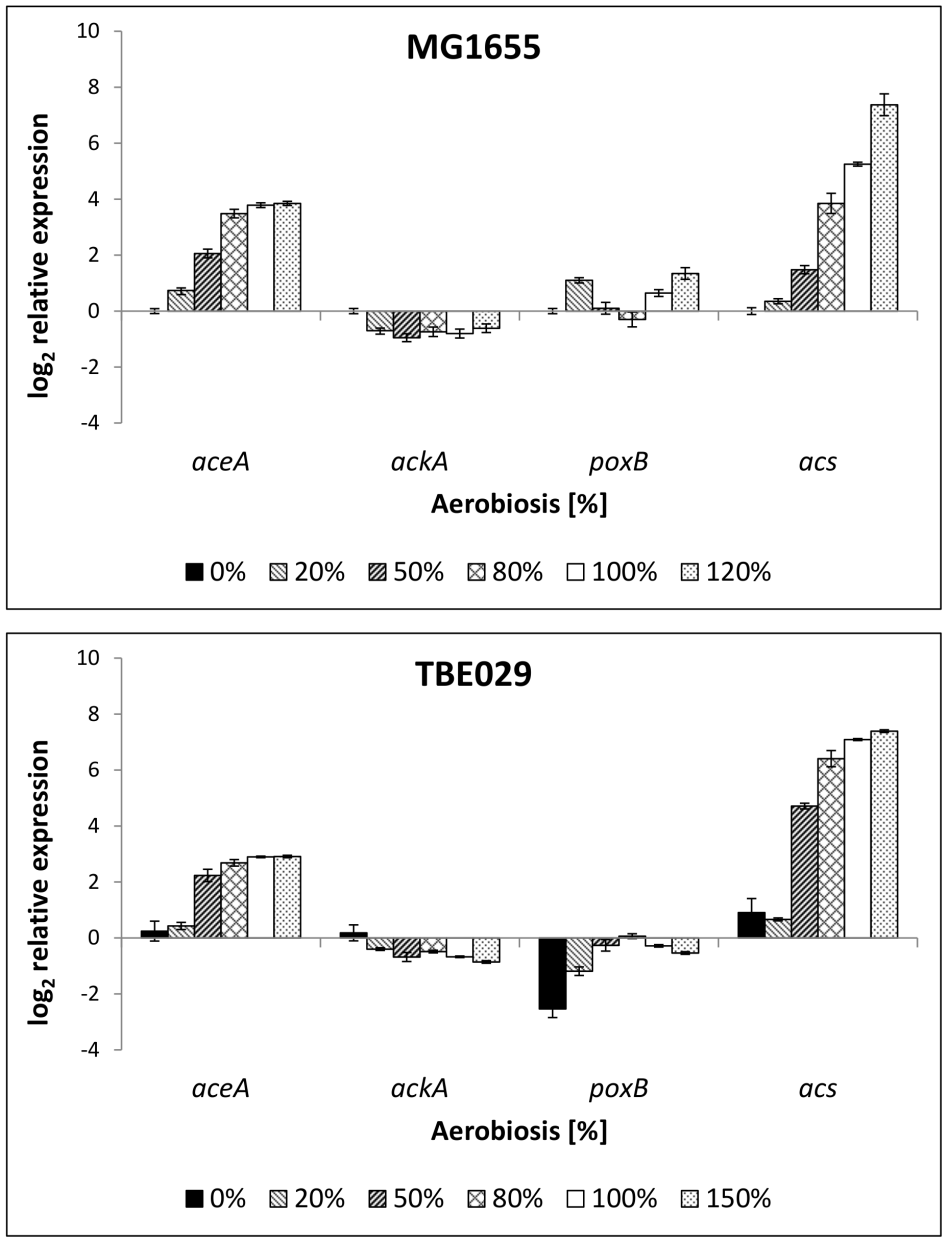
Relative expression of genes coding for enzymes of acetate metabolism in *E. coli* MG1655 (4a) and mutant strain TBE029 (4b). The transcription pattern was analyzed in cells from glucose-limited continuous cultures and normalized to the reference genes *recA, rpoD* and *ybhC* and to the expression under anaerobic conditions. *aceA*: isocitrate lyase, *ackA*: acetate kinase, *poxB*: pyruvate oxidase, *acs*: acetyl-CoA synthetase.

Under anaerobic conditions or during aerobic overflow metabolism, a part of the produced acetyl-CoA is converted to acetylphosphate by the enzyme phosphate acetyltransferase (encoded by *pta*). This in turn can be converted to acetate by the acetate kinase (gene: *ackA*) and acetate is excreted. While this pathway is reversible and the enzymes are constitutively expressed, extracellular acetate can also be activated to acetyl-CoA by acetyl-CoA synthetase (*acs*); an inducible enzyme. This reaction is functioning mainly in scavenging acetate, because of its high affinity at low extracellular concentrations [56]. Its expression is controlled in dependence of carbon metabolism (cAMP.CRP), oxygen availability (Fnr) and acetate metabolism [57]. Pyruvate oxidase (*poxB*) converts pyruvate to acetate and carbon dioxide while reducing ubiquinone. The enzyme isocitrate lyase (encoded by *aceA*) is the first enzyme of the glyoxylate bypass, converting isocitrate to succinate and glyoxylate. The glyoxylate bypass is necessary for cells growing on acetate. While expression of *aceA* and *acs* is enhanced with increasing aerobiosis in MG1655, *ackA* and *poxB* do not show dependency on oxygen availability (Fig. 5a). Although *aceA* expression still rose over the aerobiosis scale in the mutant strain, the increase in expression was a little lower than in the wild-type strain (Fig. 5b). *PoxB* expression was reduced anaerobically; *acs* expression was significantly enhanced in the microaerobic range in strain TBE029. Again in contrast to the gradual increase of *aceA* and *acs* expression over aerobiosis in MG1655, a steplike increase of expression from 20% to 50% was observed in the mutant strain.

**Figure 6:**
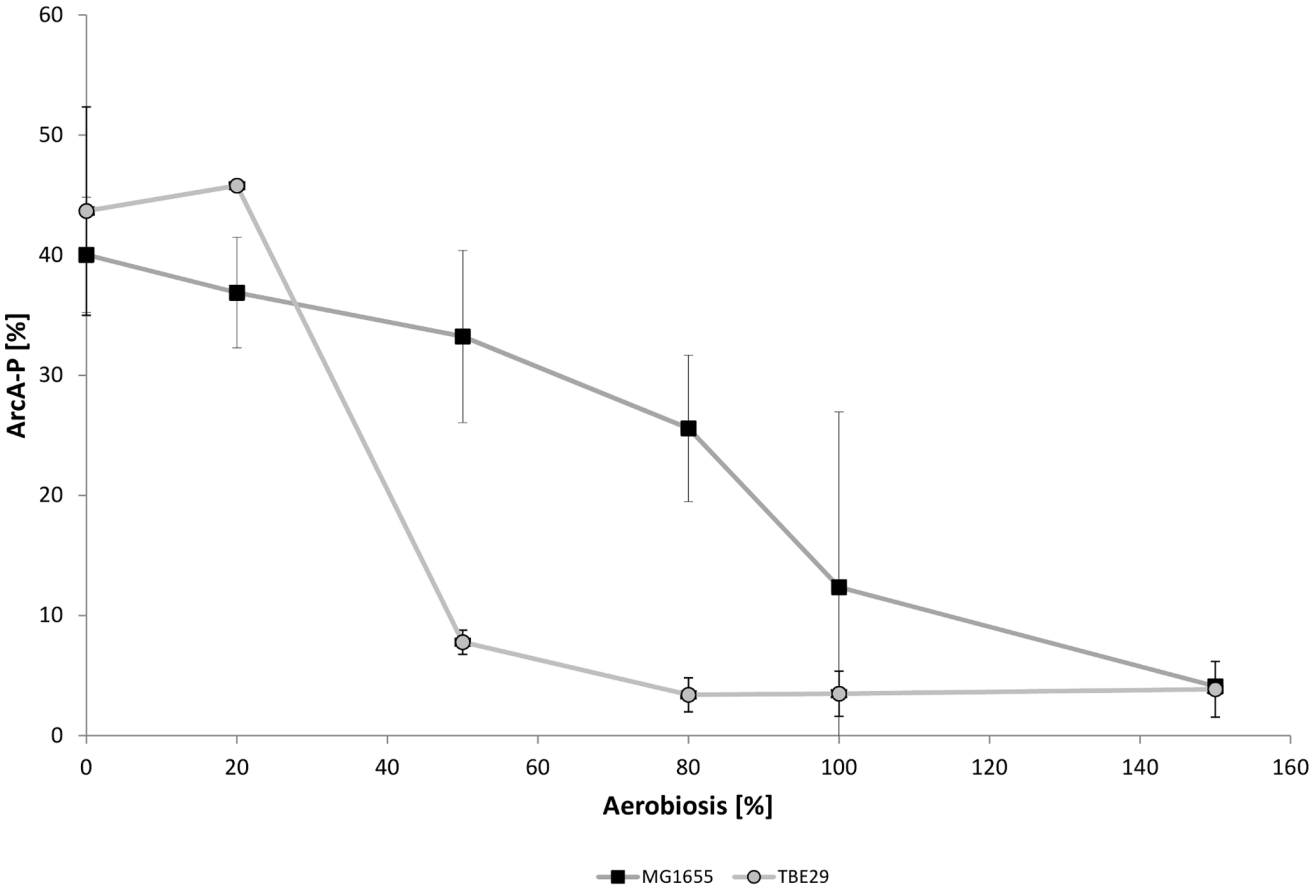
ArcA phosphorylation in *E. coli* MG1655 and mutant strain TBE029 over the aerobiosis. TBE029: Δ*nuoB*. A few data points do not seem to fit and might only be measuring inaccuracies. Nevertheless all values are displayed here as it is difficult to distinguish between measurement errors and variance in ArcA phosphorylation.

The global two-component signal transduction system ArcBA is regulating gene expression in dependence of oxygen availability, especially important under microaerobic conditions. To determine, if ArcA might be responsible for the altered gene expression profiles, ArcA activity, measurable via its phosphorylation level, was analyzed. Phosphorylated ArcA represses genes for aerobic metabolism like TCA cycle enzymes and activates genes for anaerobic metabolism like *cyd* and *ndh* [35], [40]. ArcA is phosphorylated under anaerobic conditions. In MG1655 the phosphorylation slowly descents between 0 and 80% aerobiosis and drops to zero above 80% aerobiosis (Fig. 6). This trend is changed in strain TBE029, a drastic reduction of ArcA phosphorylation occurs at lower oxygen levels, between 20 and 50% aerobiosis. The phosphorylation pattern across the aerobiosis therefore followed a sigmoidal function. The changes in ArcA phosphorylation reflect the changes in gene expression pattern. The sigmoidal decrease in ArcA phopshorylation with increasing aerobiosis as observed for TBE029 reflects the step-like increase of gene expression of *cyoA* and the TCA genes, compared to a more hyperbolic trend of ArcA phosphorylation coupled to gradual increase in gene expression for MG1655. A modified ArcA activity might hence be the reason for the gene expression patterns observed.

### Characterization of strains with a linear electron transport chain

The expression of the oxidases is influenced by the oxygen availability, with cytochrome *bo* showing maximal expression under aerobic conditions, *bd-I* under microaerobic and *bd-II* under anaerobic conditions (Fig. 3a). To analyze the significance of the three terminal oxidases, deletion strains were constructed, owning only one of the quinol oxidases in the background of the NDH I deletion strain. These strains possess a linear respiratory chain instead of a branched.

#### TBE031: cytochrome *bo* as sole terminal oxidase

**Figure 7:**
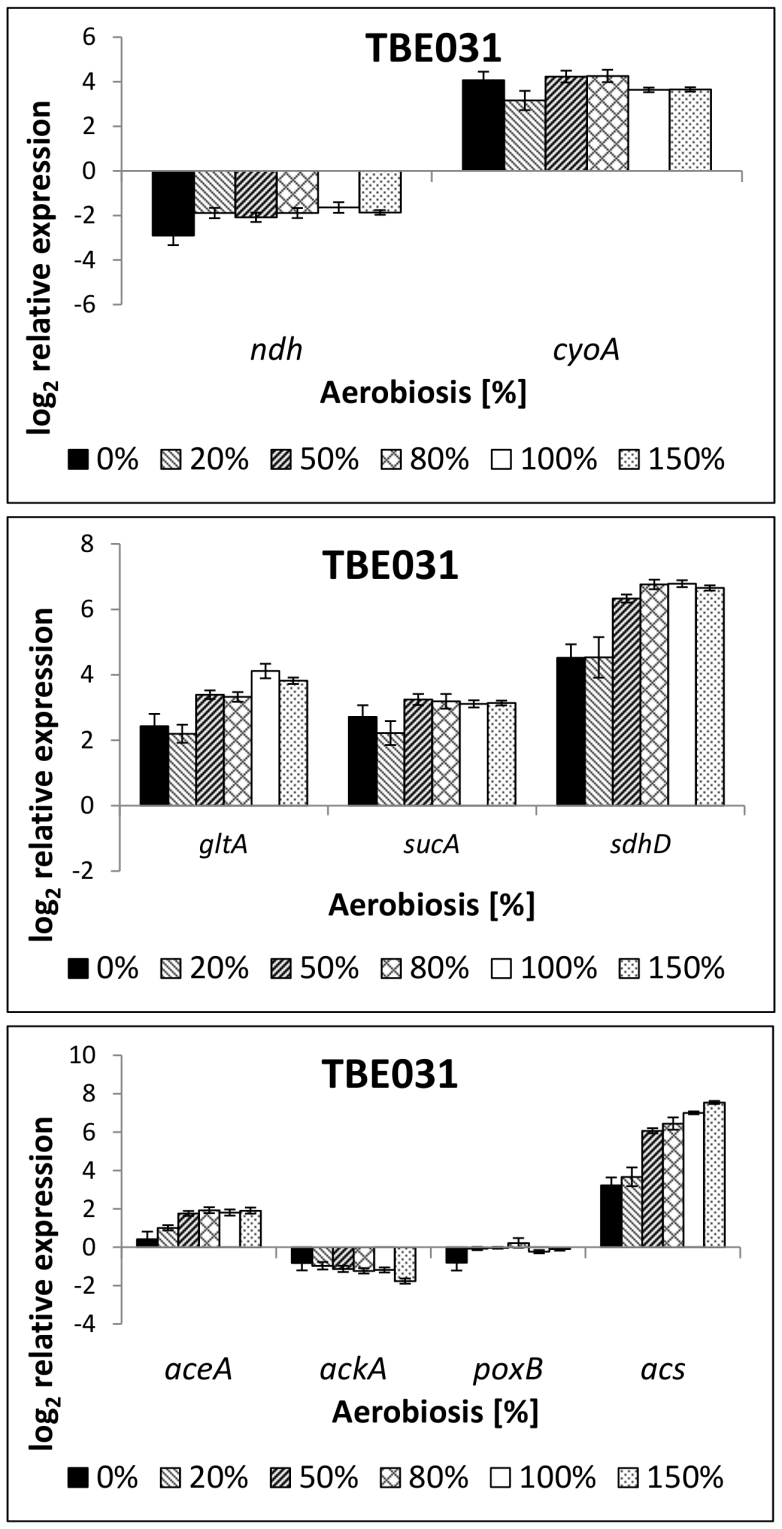
Relative expression of genes coding for respiratory enzymes (6a) and TCA cycle enzymes (6b) in strain TBE031 (Cyt *bo*). TBE031: Δ*nuoB*, Δ*cydB*, Δ*appB*. The transcription pattern was analyzed in cells from glucose-limited continuous cultures and normalized to the reference genes *recA, rpoD* and *ybhC* and to the expression of the wild-type strain under anaerobic conditions. *ndh*: NADH dehydrogenase II, *cyoA*: subunit of cytochrome oxidase *bo, gltA*: citrate synthase, *sucA*: subunit of 2-oxoglutarate dehydrogenase, *sdhD*: subunit of succinate dehydrogenase.

**Figure 8:**
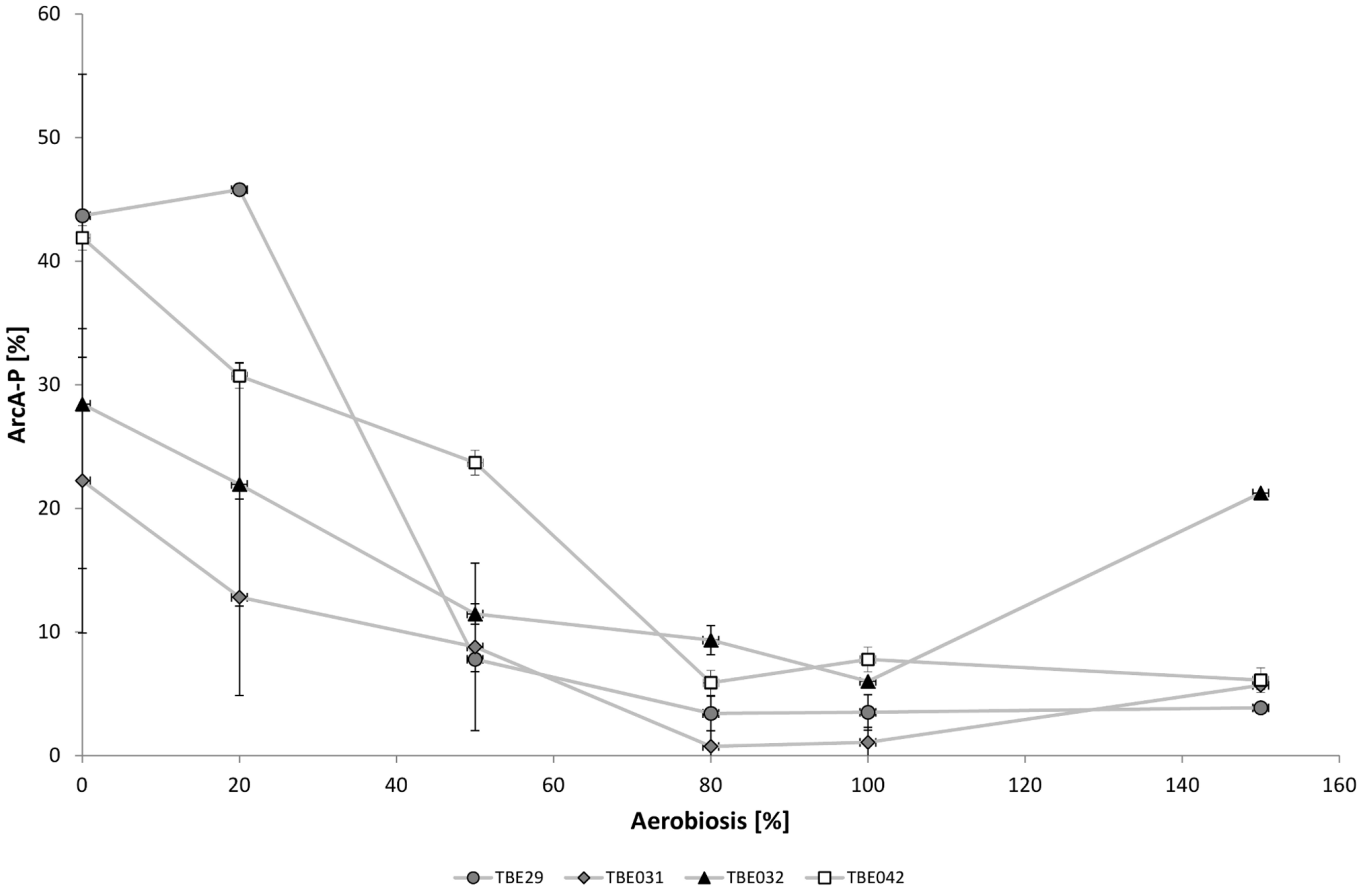
ArcA phosphorylation in *E. coli* respiratory mutant strains over the aerobiosis. TBE029: Δ*nuoB*; TBE031: Δ*nuoB*, Δ*cydB*, Δ*appB*; TBE032: Δ*nuoB*, Δ*cydB*, Δ*cyoB*; TBE042: Δ*nuoB*, Δ*cyoB*, Δ*appB*.

Strain TBE031 exhibited nearly the same acetate formation rates as TBE029; only at 50% aerobiosis acetate production was lowered. Notably, significant ethanol formation occurred even at 100% aerobiosis (Table 4). Concentrations of the other by-products were similar to those formed by strain TBE029 (not shown). In TBE031, the expression of the remaining terminal oxidase cytochrome oxidase *bo* was strongly up-regulated under anaerobic and microaerobic conditions (Fig. 7a), the same holds for genes coding for TCA cycle enzymes (Fig. 7b). Their expression at all aerobiosis levels tested was comparable to those in TBE029 under aerobic conditions. No changes in *ndh* expression were observable compared to strain TBE029. Regarding the transcription of genes important for acetate metabolism (Fig. 7c), *aceA* expression increased gradually over the aerobiosis, but less steep than in the wild-type or TBE029; *acs* expression was enhanced under anaerobic and microaerobic conditions. Figure 8 shows ArcA phosphorylation for all mutant strains: in TBE031 ArcA phosphorylation was significantly reduced an- and microaerobically.

**Table 4:** Ethanol formation rates from glucose-limited chemostat cultures at different aerobiosis levels.

	Ethanol formation rate [mmol*g^−1^*h^−1^]			
Aerobiosis [%]	TBE029	TBE031	TBE032	TBE042
0	6,62±0,27	7,05±0,50	6,00±0,31	6,82±0,25
20	2,76±0,59	3,48±0,19	2,86±0,08	3,69±0,32
50	0,89±0,10	1,39±0,87	0,10±0,08	0,96±0,90
80	0,09±0,01	0,37±0,21	0,10±0,07	0,12±0,01
100	0,02±0,03	0,45±0,08	0,07±0,03	0,05±0,00
150	0,12±0,05	0,19±0,06	#DIV/0!	0,07±0,06

By-product formation rates are calculated per hour and gram dry cell weight [mmol*g^−1^*h^−1^]. TBE029: Δ*nuoB*; TBE031: Δ*nuoB*, Δ*cydB*, Δ*appB*; TBE032: Δ*nuoB*, Δ*cydB*, Δ*cyoB*; TBE042: Δ*nuoB*, Δ*cyoB*, Δ*appB*.

As was expected from the expression of cytochrome *bo*, strain TBE031 differed from TBE029 mainly at low oxygen availabilities. Interestingly, also under completely anaerobic conditions (without external electron acceptor) differences were observable, although the respiratory chain should not be active/important.

#### TBE032: cytochrome *bd-II* as sole terminal oxidase

**Figure 9:**
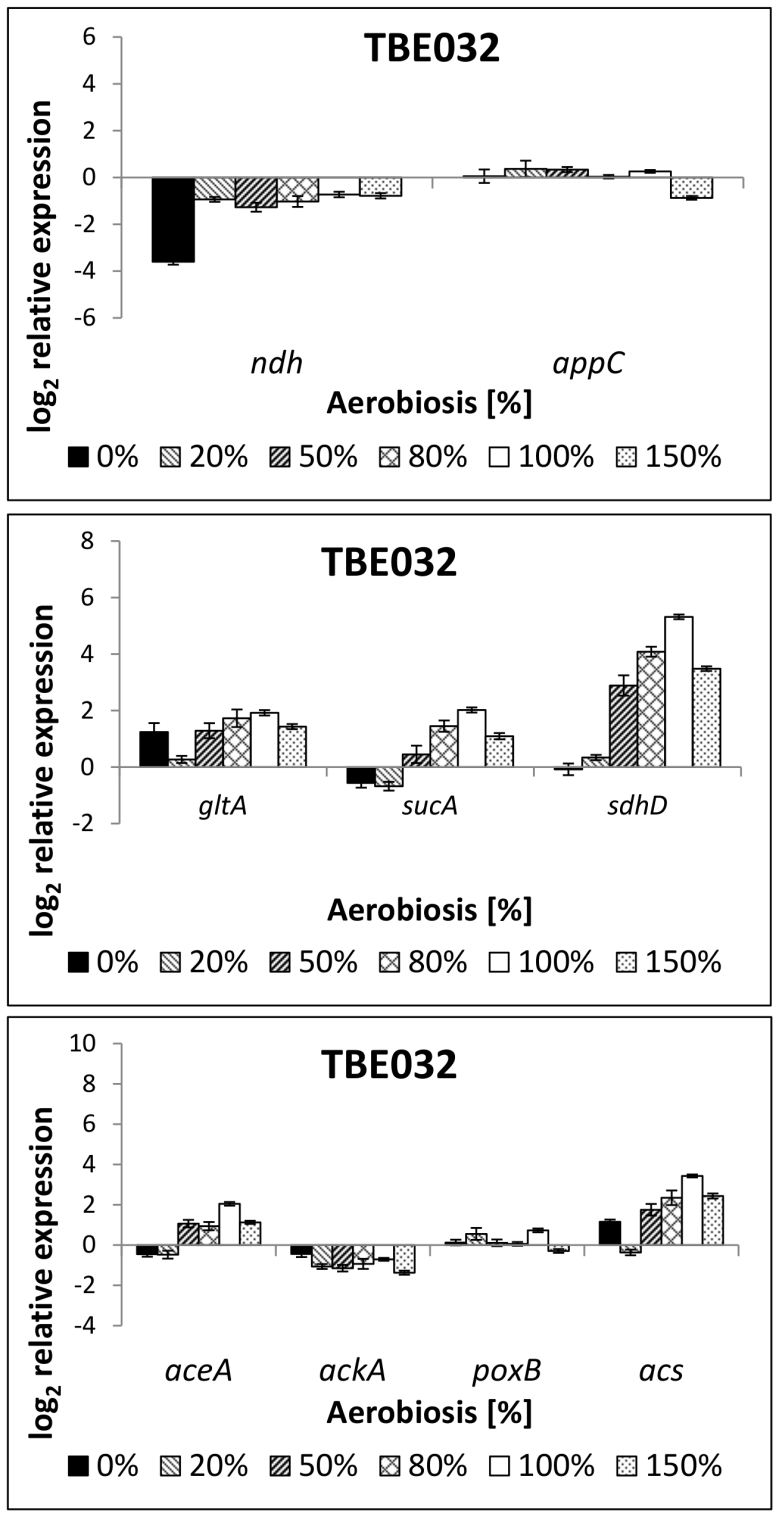
Relative expression of genes coding for respiratory enzymes (8a) and TCA cycle enzymes (8b) in strain TBE032 (Cyt *bd-II*). TBE032: Δ*nuoB*, Δ*cyoB*, Δ*cydB*. The transcription pattern was analyzed in cells from glucose-limited continuous cultures and normalized to the reference genes *recA, rpoD* and *ybhC* and to the expression of the wild-type strain under anaerobic conditions. *ndh*: NADH dehydrogenase II, *appC*: subunit of cytochrome oxidase *bd-II, gltA*: citrate synthase, *sucA*: subunit of 2-oxoglutarate dehydrogenase, *sdhD*: subunit of succinate dehydrogenase.

Strains with only cytochrome *bd-II* or cytochrome *bd-I* excreted even higher amounts of acetate at high oxygen availabilities (Table 3), strain TBE032 (Cyt *bd-II*) displayed the highest by-product formation of all strains tested under aerobic and even microaerobic conditions. The amount of acetate excreted under aerobic conditions corresponded to the level at 60% aerobiosis of the wild-type. Enhanced acetate formation occurred under all oxygen availabilities tested except for 0% aerobiosis. Ethanol concentrations were like in the parent strain TBE029. Even though enhanced acetate formation under aerobic conditions has already been described by Bekker et al. [22], they also discovered lactate excretion which we did not observe. Reasons for this discrepancy might be the different strain background, dilution rate or glucose concentration, which could influence the energetic demand. Expression of cytochrome *bd-II*, the remaining quinol oxidase in strain TBE032, did not vary over the aerobiosis except at 0% aerobiosis (Fig. 9a), transcription of *ndh* was comparable to TBE029, containing all cytochrome oxidases. The strong up-regulation of TCA cycle enzymes with increasing aerobiosis as observed in the wild-type and in TBE29, was less distinct (Fig. 9b). Regarding the acetate metabolism, strain TBE032 showed a reduced expression of isocitrate lyase, the first enzyme of the glyoxylate bypass and of acetyl-CoA synthetase, the enzyme for assimilation of acetate (Fig. 9c). While ArcA activity seemed reduced in the low microaerobic range (20% aerobiosis) compared to its parent strain TBE029, at 50% aerobiosis the phosphorylation pattern of the transcription factor was slightly enhanced and did not decrease further but stayed at a level of about 10–20% phosphorylation even under aerobic conditions.

While under anaerobic conditions by-product formation and gene expression did not vary significantly in TBE032 compared to its parent strain TBE029, at 20% aerobiosis and higher oxygen availabilities metabolic changes were observable. Even at low oxygen availabilities the respiratory chain seemed impaired, the TCA cycle less active and the fermentation pathway (acetate excretion) more active.

#### TBE042: cytochrome *bd-I* as sole terminal oxidase

**Figure 10:**
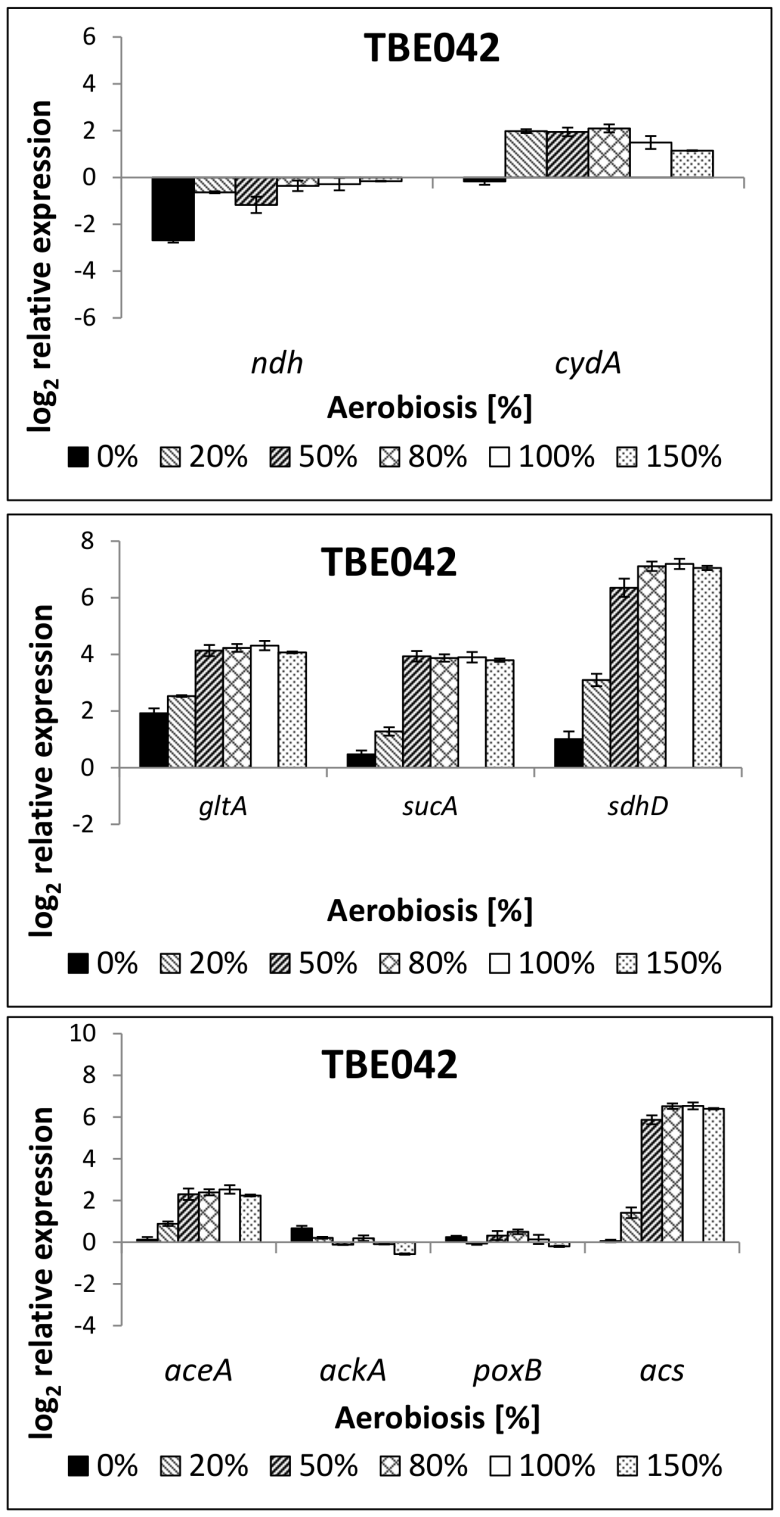
Relative expression of genes coding for respiratory enzymes (9a) and TCA cycle enzymes (9b) in strain TBE042 (Cyt *bd-I*). TBE042: Δ*nuoB*, Δ*cyoB*, Δ*appB*. The transcription pattern was analyzed in cells from glucose-limited continuous cultures and normalized to the reference genes *recA, rpoD* and *ybhC* and to the expression of the wild-type strain under anaerobic conditions. *ndh*: NADH dehydrogenase II, *cydA*: subunit of cytochrome oxidase *bd-I, gltA*: citrate synthase, *sucA*: subunit of 2-oxoglutarate dehydrogenase, *sdhD*: subunit of succinate dehydrogenase.

The third examined strain (TBE042) also excreted high amounts of acetate under aerobic conditions (Table 3), but this behavior was less distinct than in strain TBE032, containing only cytochrome oxidase *bd-II*. For the other by-products formed, no differences to TBE029, the strain with all terminal oxidases, were observable except at 50% aerobiosis, where enhanced ethanol excretion occurred. In the wild-type, cytochrome oxidase *bd-I* showed the highest expression under microaerobic conditions. Transcription of cytochrome *bd-I* was slightly up-regulated in the low microaerobic range and under aerobic conditions in this mutant strain compared to the parent strain, showing a relatively stable expression over the aerobiosis (Fig. 10a). Nevertheless, expression was lower than in the wild-type MG1655. Transcription of *ndh* was further enhanced under aerobic conditions compared to strain TBE029. The transcription of genes coding for TCA cycle enzymes was enhanced in the low microaerobic range (Fig. 10b). Like in strain TBE031 (Cyt *bo*), only minor differences in the expression of genes coding for enzymes of the acetate metabolism were observable (Fig. 10c). Under aerobic conditions, ArcA phosphorylation was enhanced like in TBE031 (Cyt *bo*), but at 50% aerobiosis, the phosphorylation level was decreased like in its parent strain TBE029 (Fig. 8).

Only minor changes in gene expression were observable in strain TBE042 compared to its parent strain TBE029, but as acetate formation rates were significantly enhanced under aerobic conditions and even at 80% aerobiosis, respiration seemed not sufficient for energy conversion at high oxygen availabilities.

## Discussion

The results obtained with strain TBE029, containing NADH dehydrogenase II as sole primary dehydrogenase yet all terminal oxidases, confirmed the prominent role of NADH dehydrogenase I under aerobic conditions, where acetate formation still occurred. Unlike expected, deletion of NADH dehydrogenase I provoked not only changes in the aerobic metabolism, but also significant differences under anaerobic conditions without external electron acceptor, where the respiratory chain should not be active. Especially genes coding for TCA cycle enzymes were upregulated. Acetate formation rates were lower under an- and microaerobic conditions, but excretion took place even under aerobic conditions. The lack of NADH dehydrogenase I led to an increased overflow under these conditions. This might also be the reason for the changed acetate production. In MG1655 in glucose-limited chemostats acetate production occurs as part of fermentative metabolism and decreases with increasing oxygen availability or respiratory metabolism, respectively. An increasing respiratory metabolism is also reflected by the glucose/O_2_ ratio (Tab. S2) that decreases with increasing aerobiosis. In the mutant strains this behavior is disturbed because the switch to respiration cannot be performed completely (also no significant differences in the glucose/O2 ratio could be detected. At low oxygen supply conditions might exist where fermentation is inhibited by oxygen but where respiratory metabolism cannot run effectively, too, due to problems in regenerating NAD^+^. This leads to acetate overflow even at glucose-limited chemostat conditions.

Particularly the activity of the transcription factor ArcA was remarkable; if phosphorylation is plotted against aerobiosis, the slope changed from hyperbolic-like for MG1655 to sigmoidal, bringing about a significantly reduced activity at 20 and 50% aerobiosis. The sudden decline in ArcA phosphorylation is reflected in the transcription rates of genes coding for enzymes of the aerobic metabolism like the TCA cycle and for cytochrome oxidase *bo*, as well as for acetate metabolism. Some genes showed in addition an enhanced anaerobic expression, reflecting a lowered phosphorylation of ArcA at 0% aerobiosis. Under aerobic conditions, the picture was not that distinct, some TCA cycle genes showed higher and some lower expression than in the wild-type strain. An about two-fold enhanced activity of NDH-II in a NDH-I deletion strain under aerobic conditions has already been shown in batch cultures [58], fitting to the doubled expression level of *ndh* measured here. Some of the differences between the wild-type strain and the NDH I deletion strain under microaerobic conditions could result from the reduced activity of ArcA, like lower acetate formation rate and enhanced transcription of cytochrome oxidase *bo* (ArcA repressed) as well as decreased expression of cytochrome oxidase *bd-I* and *bd-II* (activated by ArcA). But as also oxygen consumption is affected by the mutation, changes in FNR activity could also account for some of the observed discrepancies.

These findings clearly indicate that NADH dehydrogenase I is necessary to obtain a complete aerobic metabolism without any by-product formation under glucose-limited conditions.

Emanating from TBE029, strains TBE031, 032 and 042 lack in addition two of the cytochrome oxidases. Thereby they possess a linear respiratory chain with only one primary dehydrogenase (NADH dehydrogenase II) and one terminal oxidase (TBE031: cytochrome *bo*, TBE032: cytochrome *bd-II*, TBE042: cytochrome *bd-I*). Gene expression data of the wild-type strain, as previously shown [4], support the hypothesis of Alexeeva et al. [3] that below 50% aerobiosis the main respiratory activity is linked to cytochrome *bd-I* and above increasingly to cytochrome *bo*. The third terminal oxidase (*bd-II*) is hypothesized to be active at very low oxygen levels [21]-[23] and is maximally transcribed at 0% and 20% aerobiosis, where the other terminal oxidases exhibit low expression levels [4]. This involvement under different oxygen availabilities could be confirmed with the here analyzed mutant strains: TBE031 (Cyt *bo*) showed differences to the wild-type mainly in the an- and microaerobic range, TBE032 (Cyt *bd-II*) under aerobic conditions and TBE042 (Cyt *bd-I*) in the low microaerobic range and at high oxygen concentrations.

Strains TBE029, TBE031 and TBE042 produced lower extracellular acetate concentrations in the microaerobic range than the wild-type strain. Gene expression analysis revealed an enhanced expression of *acs* under these conditions. In strain TBE032, high amounts of acetate were measurable and *acs* expression was quite low. The activity of acetyl-CoA synthetase (Acs) is mainly anabolic to assimilate extracellular acetate [56]. In chemostat cultures, acetate production occurs not only at high dilution rates, but the acetate produced is directly assimilated via Acs [59]. Therefore, it might be speculated that acetate formation in these mutants took place like in the wild-type strain, but that acetate, produced via the Pta-AckA pathway, is directly converted to acetyl-CoA by Acs. Especially in strain TBE031 (Cyt *bd-II*), a significantly up-regulated *acs* expression might indicate the enhanced assimilation of acetate, converted via acetyl-CoA to ethanol, which is indeed increased in this strain, allowing the cells to oxidize NADH. Although the transcription of *poxB* did not vary much with the oxygen availability in the mutant strains, acetate formation might also occur via the pyruvate oxidase, which couples the oxidation of pyruvate to the reduction of ubiquinone.

The lack of NADH dehydrogenase I, together with the deletion of cytochrome *bo*, the only terminal oxidase leading to the generation of the proton motive force, resulted in severe changes in the aerobic metabolism. The energy yield per oxygen molecule is not maximal in these strains [22], higher glucose consumption rates (4.4 mmol*g^−1^*h^−1^ in TBE032 (Cyt *bd-II*), respectively 3.9 mmol*g^−1^*h^−1^ in TBE042 (Cyt *bd-I*), versus 3 mmol*g^−1^*h^−1^ in the wild-type strain and 3.1 mmol*g^−1^*h^−1^ in TBE029 and 031) and enhanced fluxes through glycolysis might therefore be necessary to meet the energetic demand. Increased respiration rates (oxygen consumption rates of 9.4 and 10.3 mmol*g^−1^*h^−1^ in TBE032 and TBE042 compared to 8.6 mmol*g^−1^*h^−1^ in the parent strain) were also observed.

Obviously the altered fluxes in metabolism are reflected in ArcA phosphorylation, confirming that ArcA is sensitive to metabolic fluxes in the cell. The phosphorylation pattern in the wild-type strain has already been shown by Rolfe et al. [55], who showed a similar activity pattern over the aerobiosis, in contrast to the results achieved by Bekker and co-workers [49]. Bekker found a maximum in activity under anaerobic conditions and in the high microaerobic range (60–80% aerobiosis) [49]. However, in their experiments the strain background (MC4100), dilution rate (0.15 h^−1^) and glucose concentration (40 mM) differed and they estimated ArcA activity via a reporter fusion. These differences might explain the diverging profiles gained for ArcA activity. Like also Rolfe and co-workers could reveal [55], a correlation could be shown between the ArcA phosphorylation level and the amount of excreted acetate. Fermentation products are known to inhibit the phosphatase activity of ArcB [34], which in turn is not able to dephosphorylate ArcA. The idea of several signals sensed by ArcB has already been discussed [55] and it seems feasible to have more than one metabolic signal integrated. The level of ArcA phosphorylation under aerobic conditions was highest in the strain which excreted most acetate (TBE032) and the lowest under microaerobic conditions in strain TBE031, who excreted minor amounts of acetate. The ArcA phosphorylation pattern in MG1655 results from the interplay of NDH-I and II, feeding electrons into the electron transport chain. Without NADH dehydrogenase I, less electrons are delivered, which has an impact on ArcB and therefore on ArcA phosphorylation and activity.

## Conclusions

Deletion of NADH dehydrogenase I caused acetate formation under conditions, where the wild-type already showed a complete aerobic metabolism without any by-product formation (100% aerobiosis) and also at higher oxygen availabilities. This effect was even enhanced in strains lacking cytochrome *bo* and one of the cytochrome *bd* oxidases, reflecting the importance of Cyt *bo* under aerobic conditions. Changes in the aerobic and microaerobic metabolism were accompanied by an altered phosphorylation pattern of the transcriptional regulator ArcA, which showed a reduced activity under microaerobic and an enhanced activity under aerobic conditions. In addition, not only aerobic respiration was influenced, but also the anaerobic metabolism, as could be shown by a reduced activity of ArcA, leading to a derepressed TCA cycle in TBE031 (Cyt *bo*).

## Supporting Information

Table S1 **Dissolved oxgen tension (pO_2_) measured in bioreactor experiments with varying oxygen supply.** The dissolved oxygen tension was determind by standard Clark's electrodes during the bioreactor experiments with defined oxygen input. As can be seen the dissolved oxygen tension was below the detection limit in all experiments performed with MG1655. On the contrast for the mutants strains dissolved oxygen was measurable for aeorobiosis values from 80%.
(DOCX)

Table S2 **Ratio of glucose uptake per O_2_ [mol/mol] used for MG1655 and the different mutant strains.** As can be seen the ratio varies with changing oxygen supply reflecting the shift from fermentative to respiratory metabolism. No obvious differences for the wildtype and the the mutant strains could be observed.
(DOCX)

## References

[1] Unden G, Bongaerts J (1997) Alternative respiratory pathways of Escherichia coli: Energetics and transcriptional regulation in response to electron acceptors. Biochim Biophys Acta 1320: 217–234. 9230919 10.1016/s0005-2728(97)00034-0

[2] Unden G, Dünnwald P (2008) The Aerobic and Anaerobic Respiratory Chain of *Escherichia coli* and *Salmonella enterica*: Enzymes and Energetics. In: Curtiss III R, Kaper JB, Squires CL, Karp PD, Neidhardt FC et al., editors. Escherichia coli and Salmonella: cellular and molecular biology. Washington, DC: ASM Press.

[3] Alexeeva S, Hellingwerf KJ, Teixeira de Mattos MJ (2002) Quantitative assessment of oxygen availability: perceived aerobiosis and its effect on flux distribution in the respiratory chain of Escherichia coli. J Bacteriol 184: 1402–1406. 11844770 10.1128/JB.184.5.1402-1406.2002 PMC134846

[4] Steinsiek S, Frixel S, Stagge S, Bettenbrock K (2011) Sumo (2011) Characterization of E. coli MG1655 and frdA and sdhC mutants at various aerobiosis levels. J Biotechnol 154: 35–45. 21458504 10.1016/j.jbiotec.2011.03.015

[5] Matsushita K, Ohnishi T, Kaback HR (1987) NADH-ubiquinone oxidoreductases of the Escherichia coli aerobic respiratory chain. Biochemistry 26: 7732–7737. 3122832 10.1021/bi00398a029

[6] Calhoun MW, Oden KL, Gennis RB, de Mattos MJ, Neijssel OM (1993) Energetic efficiency of Escherichia coli: effects of mutations in components of the aerobic respiratory chain. J Bacteriol 175: 3020–3025. 8491720 10.1128/jb.175.10.3020-3025.1993 PMC204621

[7] Bogachev AV, Murtazina RA, Skulachev VP (1996) H+/e(-) stoichiometry for NADH dehydrogenase I and dimethyl sulfoxide reductase in anaerobically grown Escherichia coli cells. J Bacteriol 178: 6233–6237. 8892824 10.1128/jb.178.21.6233-6237.1996 PMC178495

[8] Bongaerts J, Zoske S, Weidner U, Unden G (1995) Transcriptional Regulation of the Proton-Translocating Nadh Dehydrogenase Genes (Nuoa-N) of Escherichia-Coli by Electron-Acceptors, Electron-Donors and Gene Regulators. Mol Microbiol 16: 521–534. 7565112 10.1111/j.1365-2958.1995.tb02416.x

[9] Neijssel OM, Demattos MJT (1994) The Energetics of Bacterial-Growth - a Reassessment. Mol Microbiol 13: 179–182. 7984099 10.1111/j.1365-2958.1994.tb00413.x

[10] Wallace BJ, Young IG (1977) Role of Quinones in Electron-Transport to Oxygen and Nitrate in Escherichia-Coli - Studies with a Ubia- Mena- Double Quinone Mutant. Biochim Biophys Acta 461: 84–100. 195602 10.1016/0005-2728(77)90071-8

[11] Au DCT, Green GN, Gennis RB (1984) Role of Quinones in the Branch of the Escherichia-Coli Respiratory-Chain That Terminates in Cytochrome-O. J Bacteriol 157: 122–125. 6317645 10.1128/jb.157.1.122-125.1984 PMC215139

[12] Shestopalov AI, Bogachev AV, Murtazina RA, Viryasov MB, Skulachev VP (1997) Aeration-dependent changes in composition of the quinone pool in Escherichia coli - Evidence of post-transcriptional regulation of the quinone biosynthesis. FEBS Lett 404: 272–274. 9119077 10.1016/s0014-5793(97)00143-9

[13] Puustinen A, Finel M, Haltia T, Gennis RB, Wikstrom M (1991) Properties of the two terminal oxidases of Escherichia coli. Biochemistry 30: 3936–3942. 1850294 10.1021/bi00230a019

[14] Borisov VB, Gennis RB, Hemp J, Verkhovsky MI (2011) The cytochrome bd respiratory oxygen reductases. Biochim Biophys Acta 1807: 1398–1413. 21756872 10.1016/j.bbabio.2011.06.016 PMC3171616

[15] Rice CW, Hempfling WP (1978) Oxygen-limited continuous culture and respiratory energy conservation in Escherichia coli. J Bacteriol 134: 115–124. 25879 10.1128/jb.134.1.115-124.1978 PMC222225

[16] Fu HA, Iuchi S, Lin EC (1991) The requirement of ArcA and Fnr for peak expression of the cyd operon in Escherichia coli under microaerobic conditions. Mol Gen Genet 226: 209–213. 1851949 10.1007/BF00273605

[17] Cotter PA, Chepuri V, Gennis RB, Gunsalus RP (1990) Cytochrome-O (Cyoabcde) and D (Cydab) Oxidase Gene-Expression in Escherichia-Coli Is Regulated by Oxygen, Ph, and the Fnr Gene-Product. J Bacteriol 172: 6333–6338. 2172211 10.1128/jb.172.11.6333-6338.1990 PMC526817

[18] Moss F (1952) The influence of oxygen tension on respiration and cytochrome a2 formation of Escherichia coli. Aust J Exp Biol Med Sci 30: 531–540. 13041661 10.1038/icb.1952.51

[19] Becker S, Vlad D, Schuster S, Pfeiffer P, Unden G (1997) Regulatory O2 tensions for the synthesis of fermentation products in Escherichia coli and relation to aerobic respiration. Arch Microbiol 168: 290–296. 9297466 10.1007/s002030050501

[20] Tseng CP, Albrecht J, Gunsalus RP (1996) Effect of microaerophilic cell growth conditions on expression of the aerobic (cyoABCDE and cydAB) and anaerobic (narGHJI, frdABCD, and dmsABC) respiratory pathway genes in Escherichia coli. J Bacteriol 178: 1094–1098. 8576043 10.1128/jb.178.4.1094-1098.1996 PMC177770

[21] Brondsted L, Atlung T (1996) Effect of growth conditions on expression of the acid phosphatase (cyx-appA) operon and the appY gene, which encodes a transcriptional activator of Escherichia coli. J Bacteriol 178: 1556–1564. 8626281 10.1128/jb.178.6.1556-1564.1996 PMC177838

[22] Bekker M, de Vries S, Ter Beek A, Hellingwerf KJ, de Mattos MJT (2009) Respiration of Escherichia coli Can Be Fully Uncoupled via the Nonelectrogenic Terminal Cytochrome bd-II Oxidase. J Bacteriol 191: 5510–5517. 19542282 10.1128/JB.00562-09 PMC2725625

[23] Atlung T, Brondsted L (1994) Role of the transcriptional activator AppY in regulation of the cyx appA operon of Escherichia coli by anaerobiosis, phosphate starvation, and growth phase. J Bacteriol 176: 5414–5422. 8071219 10.1128/jb.176.17.5414-5422.1994 PMC196729

[24] Dassa J, Fsihi H, Marck C, Dion M, Kiefferbontemps M, et al (1991) A New Oxygen-Regulated Operon in Escherichia-Coli Comprises the Genes for a Putative 3rd Cytochrome-Oxidase and for Ph 2.5 Acid-Phosphatase (Appa). Mol Gen Genet 229: 341–352. 1658595 10.1007/BF00267454

[25] Sharma P, de Mattos MJT, Hellingwerf KJ, Bekker M (2012) On the function of the various quinone species in Escherichia coli. FEBS J 279: 3364–3373. 22521170 10.1111/j.1742-4658.2012.08608.x

[26] Unden G, Becker S, Bongaerts J, Holighaus G, Schirawski J, et al (1995) O-2-Sensing and O-2-Dependent Gene-Regulation in Facultatively Anaerobic-Bacteria. Arch Microbiol 164: 81–90. 8588737

[27] Lambden PR, Guest JR (1976) Mutants of Escherichia-Coli-K12 Unable to Use Fumarate as an Anaerobic Electron-Acceptor. J Gen Microbiol 97: 145–160. 796407 10.1099/00221287-97-2-145

[28] Kang YS, Weber KD, Yu Q, Kiley PJ, Blattner FR (2005) Genome-wide expression analysis indicates that FNR of Escherichia coli K-12 regulates a large number of genes of unknown function. J Bacteriol 187: 1135–1160. 15659690 10.1128/JB.187.3.1135-1160.2005 PMC545700

[29] Green J, Carter E, Murphy DM (2009) Interaction of molecular oxygen with oxygen vacancies on reduced TiO2: Site specific blocking by probe molecules. Chem Phys Lett 477: 340–344.

[30] Iuchi S, Lin ECC (1988) Arca (Dye), a Global Regulatory Gene in Escherichia-Coli Mediating Repression of Enzymes in Aerobic Pathways. Proc Natl Acad Sci U S A 85: 1888–1892. 2964639 10.1073/pnas.85.6.1888 PMC279886

[31] Iuchi S, Cameron DC, Lin ECC (1989) A 2nd Global Regulator Gene (Arcb) Mediating Repression of Enzymes in Aerobic Pathways of Escherichia-Coli. J Bacteriol 171: 868–873. 2644240 10.1128/jb.171.2.868-873.1989 PMC209676

[32] Gunsalus RP, Park SJ (1994) Aerobic-anaerobic gene regulation in Escherichia coli: control by the ArcAB and Fnr regulons. Res Microbiol 145: 437–450. 7855430 10.1016/0923-2508(94)90092-2

[33] Iuchi S, Lin ECC (1992) Purification and Phosphorylation of the Arc Regulatory Components of Escherichia-Coli. J Bacteriol 174: 5617–5623. 1512197 10.1128/jb.174.17.5617-5623.1992 PMC206507

[34] Iuchi S (1993) Phosphorylation Dephosphorylation of the Receiver Module at the Conserved Aspartate Residue Controls Transphosphorylation Activity of Histidine Kinase in Sensor Protein Arcb of Escherichia-Coli. J Biol Chem 268: 23972–23980. 8226939

[35] Liu XQ, De Wulf P (2004) Probing the ArcA-P modulon of Escherichia coli by whole genome transcriptional analysis and sequence recognition profiling. J Biol Chem 279: 12588–12597. 14711822 10.1074/jbc.M313454200

[36] Salmon KA, Hung S, Steffen NR, Krupp R, Baldi P, et al (2005) Global gene expression profiling in Escherichia coli K12 - Effects of oxygen availability and ArcA. J Biol Chem 280: 15084–15096. 15699038 10.1074/jbc.M414030200

[37] Salmon K, Hung SP, Mekjian K, Baldi P, Hatfield GW, et al (2003) Global gene expression profiling in Escherichia coli K12 - The effects of oxygen availability and FNR. J Biol Chem 278: 29837–29855. 12754220 10.1074/jbc.M213060200

[38] Shalel-Levanon S, San KY, Bennett GN (2005) Effect of oxygen, and ArcA and FNR regulators on the expression of genes related to the electron transfer chain and the TCA cycle in Escherichia coli. Metab Eng 7: 364–374. 16140031 10.1016/j.ymben.2005.07.001

[39] Lazazzera BA, Beinert H, Khoroshilova N, Kennedy MC, Kiley PJ (1996) DNA binding and dimerization of the Fe-S-containing FNR protein from Escherichia coli are regulated by oxygen. J Biol Chem 271: 2762–2768. 8576252 10.1074/jbc.271.5.2762

[40] Lynch AS, Lin ECC (1996) Transcriptional control mediated by the ArcA two-component response regulator protein of Escherichia coli: Characterization of DNA binding at target promoters. J Bacteriol 178: 6238–6249. 8892825 10.1128/jb.178.21.6238-6249.1996 PMC178496

[41] Alexeeva S, Hellingwerf KJ, de Mattos MJT (2003) Requirement of ArcA for redox regulation in Escherichia coli under microaerobic but not anaerobic or aerobic conditions. J Bacteriol 185: 204–209. 12486057 10.1128/JB.185.1.204-209.2003 PMC141817

[42] Alexeeva S, de Kort B, Sawers G, Hellingwerf KJ, de Mattos MJ (2000) Effects of limited aeration and of the ArcAB system on intermediary pyruvate catabolism in Escherichia coli. J Bacteriol 182: 4934–4940. 10940038 10.1128/jb.182.17.4934-4940.2000 PMC111374

[43] Levanon SS, San KY, Bennett GN (2005) Effect of oxygen on the Escherichia coli ArcA and FNR regulation systems and metabolic responses. Biotechnol Bioeng 89: 556–564. 15669087 10.1002/bit.20381

[44] Iuchi S, Chepuri V, Fu HA, Gennis RB, Lin ECC (1990) Requirement for Terminal Cytochromes in Generation of the Aerobic Signal for the Arc Regulatory System in Escherichia-Coli - Study Utilizing Deletions and Lac Fusions of Cyo and Cyd. J Bacteriol 172: 6020–6025. 2170337 10.1128/jb.172.10.6020-6025.1990 PMC526924

[45] Georgellis D, Kwon O, Lin EC (2001) Quinones as the redox signal for the arc two-component system of bacteria. Science 292: 2314–2316. 11423658 10.1126/science.1059361

[46] Malpica R, Franco B, Rodriguez C, Kwon O, Georgellis D (2004) Identification of a quinone-sensitive redox switch in the ArcB sensor kinase. Proc Natl Acad Sci U S A 101: 13318–13323. 15326287 10.1073/pnas.0403064101 PMC516565

[47] Georgellis D, Kwon O, Lin EC (1999) Amplification of signaling activity of the arc two-component system of Escherichia coli by anaerobic metabolites. An in vitro study with different protein modules. J Biol Chem 274: 35950–35954. 10585483 10.1074/jbc.274.50.35950

[48] Rodriguez C, Kwon O, Georgellis D (2004) Effect of D-lactate on the physiological activity of the ArcB sensor kinase in Escherichia coli. J Bacteriol 186: 2085–2090. 15028693 10.1128/JB.186.7.2085-2090.2004 PMC374410

[49] Bekker M, Alexeeva S, Laan W, Sawers G, de Mattos JT, et al (2010) The ArcBA Two-Component System of Escherichia coli Is Regulated by the Redox State of both the Ubiquinone and the Menaquinone Pool. J Bacteriol 192: 746–754. 19933363 10.1128/JB.01156-09 PMC2812447

[50] Alvarez AF, Rodriguez C, Georgellis D (2013) Ubiquinone and Menaquinone Electron Carriers Represent the Yin and Yang in the Redox Regulation of the ArcB Sensor Kinase. J Bacteriol 195: 3054–3061. 23645604 10.1128/JB.00406-13 PMC3697540

[51] Baba T, Ara T, Hasegawa M, Takai Y, Okumura Y, et al (2006) Construction of Escherichia coli K-12 in-frame, single-gene knockout mutants: the Keio collection. Mol Syst Biol 2. 10.1038/msb4100050 PMC1681482 16738554

[52] Datsenko KA, Wanner BL (2000) One-step inactivation of chromosomal genes in Escherichia coli K-12 using PCR products. Proc Natl Acad Sci U S A 97: 6640–6645. 10829079 10.1073/pnas.120163297 PMC18686

[53] Evans C, Herbert D, Tempest D (1970) Chapter XIII The Continuous Cultivation of Micro-organisms2. Construction of a Chemostat. Meth Microbiol 2: 277–327.

[54] Hellemans J, Mortier G, De Paepe A, Speleman F, Vandesompele J (2007) qBase relative quantification framework and software for management and automated analysis of real-time quantitative PCR data. Genome Biol 8: R19. 17291332 10.1186/gb-2007-8-2-r19 PMC1852402

[55] Rolfe MD, Ter Beek A, Graham AI, Trotter EW, Asif HMS, et al (2011) Transcript Profiling and Inference of Escherichia coli K-12 ArcA Activity across the Range of Physiologically Relevant Oxygen Concentrations. J Biol Chem 286: 10147–10154. 21252224 10.1074/jbc.M110.211144 PMC3060466

[56] Brown TDK, Jonesmortimer MC, Kornberg HL (1977) Enzymic Interconversion of Acetate and Acetyl-Coenzyme-a in Escherichia-Coli. J Gen Microbiol 102: 327–336. 21941 10.1099/00221287-102-2-327

[57] Kumari S, Beatty CM, Browning DF, Busby SJW, Simel EJ, et al (2000) Regulation of acetyl coenzyme A synthetase in Escherichia coli. J Bacteriol 182: 4173–4179. 10894724 10.1128/jb.182.15.4173-4179.2000 PMC101899

[58] Kihira C, Hayashi Y, Azuma N, Noda S, Maeda S, et al (2012) Alterations of glucose metabolism in Escherichia coli mutants defective in respiratory-chain enzymes. J Biotechnol 158: 215–223. 21740932 10.1016/j.jbiotec.2011.06.029

[59] Renilla S, Bernal V, Fuhrer T, Castano-Cerezo S, Pastor JM, et al (2012) Acetate scavenging activity in Escherichia coli: interplay of acetyl-CoA synthetase and the PEP-glyoxylate cycle in chemostat cultures. Appl Microbiol Biotechnol 93: 2109–2124. 21881893 10.1007/s00253-011-3536-4

