# Supplementary material for: Analysis of Escherichia coli Mutants with a Linear Respiratory Chain
Source: PLoS One. 2014 Jan 27;9(1):e87307. doi: 10.1371/journal.pone.0087307 (PMC3903629; doi:10.1371/journal.pone.0087307)
Supplement: Table S1 — Dissolved oxgen tension (pO2) measured in bioreactor experiments with varying oxygen supply. The dissolved oxygen tension was determind by standard Clark's electrodes during the bioreactor experiments with defined oxygen input. As can be seen the dissolved oxygen tension was below the detection limit in all experiments performed with MG1655. On the contrast for the mutants strains dissolved oxygen was measurable for aeorobiosis values from 80%. (DOCX) [file pone.0087307.s001.docx]

**Table S1: Dissolved oxgen tension (pO_2_) measured in bioreactor experiments with varying oxygen supply.**

The dissolved oxygen tension was determind by standard Clark´s electrodes during the bioreactor experiments with defined oxygen input. As can be seen the dissolved oxygen tension was below the detection limit in all experiments performed with MG1655. On the contrast for the mutants strains dissolved oxygen was measurable for aeorobiosis values from 80%.

| strain/  aerobiosis [%] | MG1655 | TBE029 | TBE031 | TBE032 | TBE042 |
| --- | --- | --- | --- | --- | --- |
| 0 | 0.0 | 0.0 | 0.0 | 0.0 | 0.0 |
| 20 | 0.0 | 0.0 | 0.0 | 0.0 | 0.0 |
| 50 | 0.0 | 0.0 | 0.0 | 0.0 | 0.0 |
| 80 | 0.0 | 1.9 | 1.9 | 2.4 | 2.0 |
| 100 | 0.0 | 5.4 | 9.5 | 8.6 | 3.6 |
| 150 |  | 17.9 | 17.4 | 24.5 | 16.0 |
