# Supplementary material for: Analysis of Escherichia coli Mutants with a Linear Respiratory Chain
Source: PLoS One. 2014 Jan 27;9(1):e87307. doi: 10.1371/journal.pone.0087307 (PMC3903629; doi:10.1371/journal.pone.0087307)
Supplement: Table S2 — Ratio of glucose uptake per O2 [mol/mol] used for MG1655 and the different mutant strains. As can be seen the ratio varies with changing oxygen supply reflecting the shift from fermentative to respiratory metabolism. No obvious differences for the wildtype and the the mutant strains could be observed. (DOCX) [file pone.0087307.s002.docx]

**Table S2**: Ratio of glucose uptake per O_2_ [mol/mol] used for MG1655 and the different mutant strains. As can be seen the ratio varies with changing oxygen supply reflecting the shift from fermentative to respiratory metabolism. No obvious differences for the wildtype and the the mutant strains could be observed

| strain/  aerobiosis [%] | MG1655 | TBE029 | TBE031 | TBE032 | TBE042 |
| --- | --- | --- | --- | --- | --- |
| 0 |  |  |  |  |  |
| 20 | 2.26 ± 0.65 | 2.64 ± 0.49 | 2.21 ± 1.57 | 1.31 ± 0.73 | 1.57 |
| 50 | 0.85 ± 0.21 | 0.73 ± 0.00 | 0.82 ± 0.45 | 0.73 ± 0.27 | 0.64 ± 0.03 |
| 80 | 0.44 ± 0.09 | 0.51 ± 0.02 | 0.45 ± 0.19 | 0.56 ± 0.05 | 0.51 ± 0.00 |
| 100 | 0.37 ± 0.05 | 0.48 ± 0.02 | 0.47 ± 0.05 | 0.34 | 0.41 ± 0.01 |
| 150 | 0.35 ± 0.06 | 0.41 | 0.4 | 0.47 | 0.38 |
